# Apparent Non-Response to Phosphodiesterase Type 5 Inhibitors in Erectile Dysfunction: A Narrative Review of Structured Reassessment and Management

**DOI:** 10.3390/medicina62081582

**Published:** 2026-08-18

**Authors:** Aris Kaltsas

**Affiliations:** Third Department of Urology, Attikon University Hospital, School of Medicine, National and Kapodistrian University of Athens, 12462 Athens, Greece; akaltsas@med.uoa.gr

**Keywords:** erectile dysfunction, phosphodiesterase type 5 inhibitors, non-response, treatment optimization, shared decision-making, narrative review

## Abstract

*Background and Objectives*: Apparent non-response to phosphodiesterase type 5 (PDE5) inhibitors is common, but the label conflates inadequate instruction or drug exposure, partial or absent response, treatment discontinuation, and coexisting clinical contributors. This narrative review presents an author-proposed structured reassessment framework, not a new or validated diagnostic classification. *Materials and Methods*: PubMed/MEDLINE was searched from database inception to 7 August 2026 using a documented, reproducible search strategy, with targeted checks of guideline, regulatory, clinical, observational, and mechanistic sources; selection was purposive, without a registered protocol, quantitative synthesis, or certainty-of-evidence grading. *Results*: PDE5 inhibitors preserve nitric oxide-cyclic guanosine monophosphate signaling but require sexual stimulation, adequate exposure, and sufficient erectile physiology. In one specialist-referral cohort, 88 of 250 men (35.2%) responded after structured re-education, being 88 of the 115 (76.5%) who accepted it, which should not be generalized. The framework separates trial and exposure adequacy, observed response documented with validated measures where feasible, targeted evaluation of contributing domains, and shared selection of oral optimization or an established non-oral option. Persistent clinically meaningful oral-PDE5-inhibitor non-response is defined by principle rather than by a fixed number of attempts, agents, or mandatory daily tadalafil. *Conclusions*: Structured reassessment may clarify why an observed response is inadequate and may support preference-sensitive management; whether it does so better than existing guidance has not been tested. Switching agents or daily tadalafil is not a prerequisite and should not delay vacuum therapy, intracavernosal injection, or penile-prosthesis discussion.

## 1. Introduction

Phosphodiesterase type 5 (PDE5) inhibitors are the first-line pharmacological therapy for most men with erectile dysfunction (ED) [1,2,3,4]. Their efficacy is well established, yet discontinuation and dissatisfaction remain common outside clinical trials [5]. A history that “the pill did not work” is therefore clinically important but insufficiently specific. It may reflect incorrect administration, inadequate drug exposure, limited adherence or access, an incomplete erectile response; adverse effects; a mismatch between treatment and patient or partner expectations; or ED associated with vascular, endocrine, neurogenic, structural, medication-related, or psychosexual factors [2,5,6,7,8].

Management pathways for apparent PDE5-inhibitor non-response are not new. For two decades, reviews and clinical guidance have emphasized checking instructions and use; assessing relevant comorbidity; optimizing an oral regimen when appropriate; and discussing mechanical, injectable, or surgical options [6,9,10,11]. A 2026 article by Pescatori et al. similarly reviewed established and emerging treatments after PDE5-inhibitor failure [12]. A contemporaneous Perspective by Hatzichristou proposed an “Erectile Reserve Test” and described PDE5 inhibitors as initiators, enablers, and sustainers of erectile function [13]; Section 7.4 sets out where the present review agrees with that proposal and where it does not. A comprehensive review of the pathophysiology, diagnosis and contemporary management of erectile dysfunction, published in April 2026, covers second-line options after an inadequate response but does not address the reassessment step itself [14]. The present review should therefore be read as an updated, pragmatic, author-proposed educational synthesis rather than as a novel classification or validated clinical instrument.

A central problem in the earlier literature is that several different conceptual axes are often combined under one label. Trial adequacy and pharmacokinetic exposure describe whether the treatment had a fair opportunity to work. Complete, partial, or absent response describes an observed outcome. Vascular, endocrine, neurogenic, structural, medication-related, and psychosexual factors describe possible contributors. Persistent oral-treatment non-response is a final treatment status, not an etiology. Keeping these axes separate avoids implying that they are mutually exclusive categories or successive stages of biological severity.

This review has four aims. First, it summarizes the mechanisms and practical conditions that determine observed PDE5-inhibitor effectiveness. Second, it organizes reassessment into four distinct stages: core assessment of trial and exposure adequacy, validated characterization of response, targeted evaluation of contributing domains, and shared treatment selection. Third, it proposes a principle-based definition of persistent clinically meaningful oral-PDE5-inhibitor non-response without mandatory thresholds for numbers of attempts, agents, or daily dosing. Fourth, it distinguishes established symptomatic treatment from hypotheses about erectile reserve, tissue preservation, and disease modification. Patient goals, partner context, and female sexual difficulties are included because treatment is experienced within a sexual relationship as well as by the individual [5,15].

Throughout this article, apparent non-response denotes the initial report that treatment was ineffective before adequacy and outcome have been characterized. Partial response and absent response denote measured outcomes. Persistent clinically meaningful oral-PDE5-inhibitor non-response denotes the final oral-treatment status defined in Section 3.2.4. The term does not imply molecular resistance and is not a stand-alone diagnosis. Table 1 summarizes the misconceptions that most often underlie the label and their evidence-aligned counterparts.

## 2. Evidence Acquisition

This is a narrative review rather than a systematic review or meta-analysis. PubMed/MEDLINE was searched from database inception to 7 August 2026 using a documented, reproducible search strategy. The core strategy combines terms for erectile dysfunction and PDE5 inhibitors with terms for non-response, treatment failure, administration, misuse, exposure, adherence, discontinuation, and treatment optimization. Targeted components address validated response measures; diabetes and cardiometabolic disease; hypogonadism; post-prostatectomy and other neurogenic ED; structural and veno-occlusive dysfunction; medication-related ED; psychosexual, relationship, partner, and female-sexual-function factors; cardiovascular and prescribing safety; vacuum erection devices; intracavernosal injection; penile prostheses; low-intensity shockwave therapy; and selected combination approaches. The complete Boolean strings, the final search date, and the record count retrieved by each query are reported in Supplementary Method S1.

Sources considered included clinical-practice guidelines, consensus statements, systematic reviews, meta-analyses, randomized trials, prospective and retrospective clinical studies, large observational safety studies, narrative and conceptual reviews, and selected mechanistic or animal studies. Preclinical evidence was used only for explicitly identified mechanistic or hypothesis-generating statements. Regulatory labels and product monographs were used for licensed indications, contraindications, dosing, and product availability. No manufacturer webpage is cited; where a product-specific statement was required, the official summary of product characteristics or product monograph was used, and never to infer clinical efficacy, acceptability, adherence, or responder conversion.

Titles, abstracts, and full texts were selected purposively for directness to the review question, study design, population relevance, sample size, recency, and consistency with current guidance. Because selection was performed within a single-author narrative-review process without duplicate screening, a registered protocol, a PRISMA flow diagram, or quantitative pooling, selection and citation bias cannot be excluded. No formal GRADE assessment was undertaken; this review therefore does not assign informal certainty grades. Published appraisals also show variability in the quality of ED practice guidelines [38].

The structured reassessment framework is an author-proposed synthesis that maps the cited literature into four conceptually distinct stages: trial/exposure adequacy, observed response, contributing clinical domains, and final management status. During revision, the author iteratively grouped the reassessment elements according to the clinical question they addressed—trial and exposure adequacy, observed response, contributing clinical domains, or management and final oral-treatment status—and reconciled this organization against the cited guidelines and prior reviews. No Delphi process, external expert consensus, patient or partner panel, content-validity exercise, or inter-rater reliability study was performed. Citations support individual components of reassessment; they do not validate the framework as a whole. During revision, bibliographic-identity checks were performed on an intermediate reference list, and 14 numerical and claim-scope checks were performed for reviewer-flagged and selected high-risk claims. These author-supervised checks were not an independent verification of the complete 104-reference original submission, the complete 119-reference revised list, or every sentence–reference pair. Use of generative artificial intelligence in preparing the figures and supporting the revision workflow is reported in Supplementary Methods S3 and S4.

## 3. Mechanistic Basis and a Working Definition of Non-Response

### 3.1. PDE5 Inhibitors Facilitate, but Do Not Initiate, Erection

Penile erection requires neural and endothelial nitric oxide (NO) release, soluble guanylyl-cyclase activation, cyclic guanosine monophosphate (cGMP) accumulation, reduction of intracellular calcium, cavernosal smooth-muscle relaxation, increased arterial inflow, and veno-occlusive trapping [16,17,18]. PDE5 is the principal cGMP-hydrolyzing phosphodiesterase in the corpus cavernosum. PDE5 inhibitors reduce cGMP degradation and thereby amplify an erectile signal that has already been initiated; they do not independently generate sexual arousal or neural NO release [16,17,19,20]. Because PDE5 inhibition amplifies an existing nitric oxide–cyclic guanosine monophosphate signal rather than generating one, a clinical response requires sufficient endothelial, neural and smooth-muscle substrate [16,17,18,19,20]. Severe endothelial impairment, neural injury, smooth-muscle loss or arterial insufficiency may therefore limit the response despite adequate drug exposure [39,40,41].

**Figure 1:**
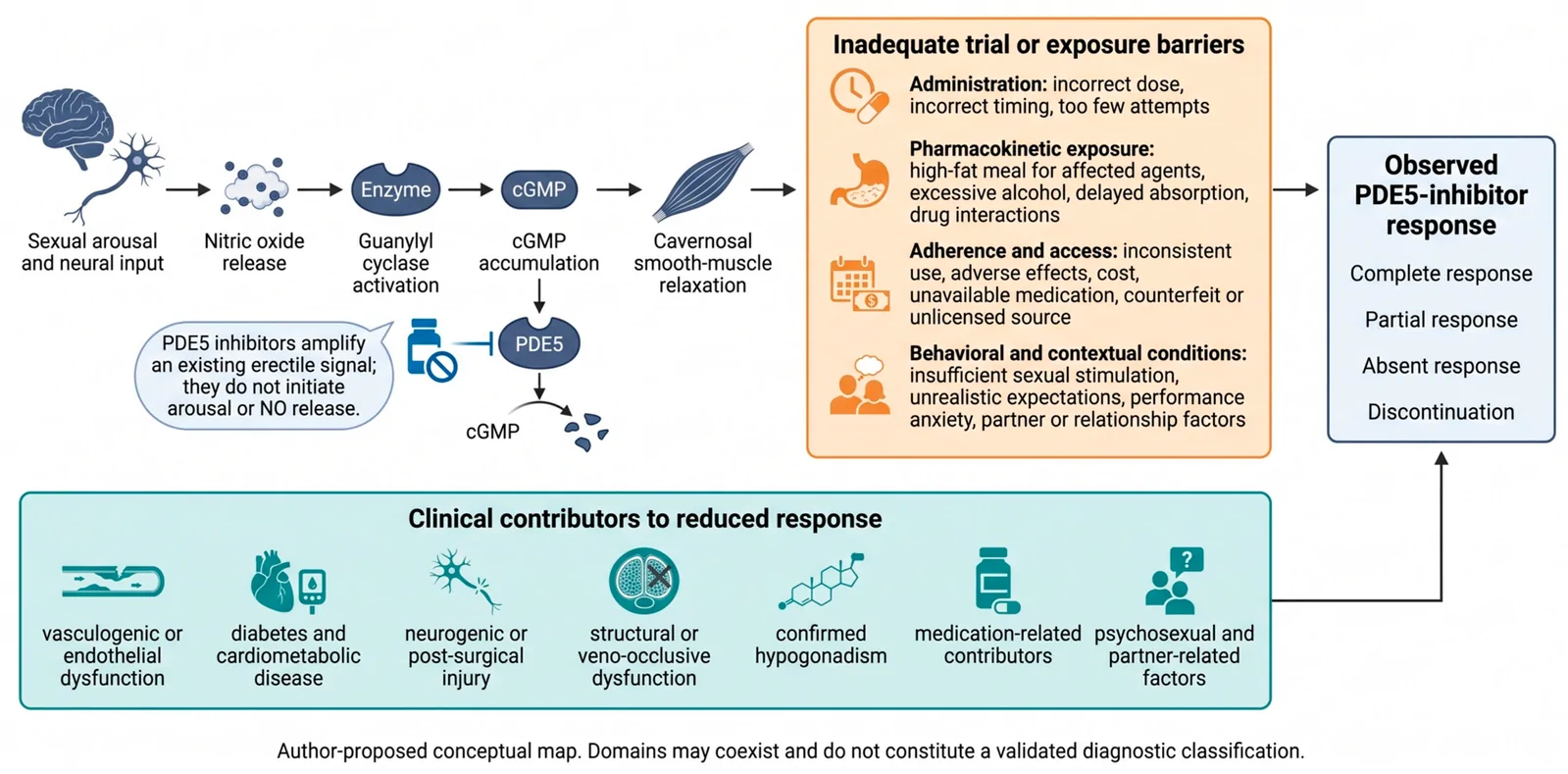
Barriers to observed PDE5-inhibitor effectiveness. PDE5 inhibition reduces cGMP degradation and therefore amplifies an erectile signal initiated by sexual arousal and neural or endothelial NO release [16,17,18,19,20]. Inadequate trial or exposure barriers—including administration, pharmacokinetic exposure, adherence and access, and behavioral or contextual conditions—are shown separately from the biological pathway and from clinical contributors to reduced response. Observed outcomes are classified as complete response, partial response, absent response, or discontinuation and should be documented with validated measures when feasible. Domains may coexist; this author-proposed conceptual map is not a validated diagnostic classification. Created in BioRender. Kaltsas, A. (2026). https://BioRender.com/798mers.

Observed effectiveness also depends on factors outside the NO–cGMP pathway. Incorrect dose or timing, too few or poor-quality attempts, food effects for susceptible agents, excessive alcohol, interacting medication, inconsistent use, adverse effects, cost, limited access, and unlicensed or counterfeit supply can all reduce effective exposure [6,7,8,42,43]. Insufficient sexual stimulation, unrealistic expectations, performance anxiety, and partner or relationship circumstances are behavioral or contextual conditions, not pharmacokinetic mechanisms [5,6,8]. Patient education is therefore necessary to achieve the expected pharmacological effect. Figure 1 separates these trial and exposure barriers from the biological pathway and from clinical contributors to reduced response.

Because the observed response is produced jointly by pharmacology, trial conditions, physiology, and context, it should not be interpreted as a specific diagnostic test. Its clinical meaning increases only after the regimen and conditions of use have been documented, and the response has been measured consistently.

### 3.2. An Author-Proposed Structured Reassessment Framework

The framework uses four sequential stages for clinical organization, but these stages are not a hierarchy of etiologies or a ladder of progressive disease severity (Table 2). Each stage answers a different question: Did the treatment have an adequate opportunity to work? What response occurred? Which factors may have contributed? Which next option best fits the patient’s goals and clinical circumstances? Domains may coexist, and movement through the framework need not be linear when safety, intolerance, severe structural or neurogenic disease, cost, access, or patient preference supports earlier use of an established second-line treatment.

#### 3.2.1. Stage 1: Trial and Exposure Adequacy

Core reassessment begins with the identity and source of the drug; dose and formulation; instructions; timing relative to food, alcohol, and intended sexual activity; sexual stimulation; number and quality of opportunities; adherence; adverse effects; cost and access; and patient and partner expectations [5,6,7,8,42,43]. These items can be organized into four subdomains: administration; pharmacokinetic exposure; adherence and supply; and behavioral or contextual conditions. This broader grouping avoids treating “false non-response” and “pharmacokinetic misuse” as separate diagnoses.

The potential benefit of reassessment is clinically relevant but should be reported with the correct denominator. In a prospective cohort of 250 men referred to a specialist andrology service with apparent PDE5-inhibitor non-response, 172 (68.8%) had at least one administration error. Of 115 men who accepted structured re-education, 88 responded. Thus, conversion occurred in 88 of 250 men in the full referred cohort (35.2%) and in 88 of 115 among the selected subgroup who accepted re-education (76.5%) [7]. These findings support careful instruction and rechallenge when acceptable to the patient; they do not show that most patients with apparent non-response are false non-responders.

#### 3.2.2. Stage 2: Characterization of the Observed Response

The response should be recorded as complete, partial, absent, or treatment discontinuation. These are outcomes, not etiological phenotypes. Whenever feasible, the assessment should use a validated measure such as the International Index of Erectile Function erectile-function domain (IIEF-EF) or Sexual Health Inventory for Men (SHIM), Erection Hardness Score (EHS), Sexual Encounter Profile questions 2 and 3 (SEP2 and SEP3), or Global Assessment Question (GAQ). An anchor-based classification has validated complete, partial, and non-response categories against other patient-reported outcomes [21]. The clinical interpretation should also consider whether the degree of improvement is meaningful to the patient and compatible with the couple’s sexual goals. Discontinuation must be analyzed separately from pharmacological non-response. In the largest meta-analysis of first-generation PDE5-inhibitor dropout, partner-related problems and perceived lack of efficacy were the leading reasons, although no single factor predominated and discontinuation usually reflected a combination of medical, psychosocial, and relational factors [5]. Perceived lack of efficacy reported at dropout is not equivalent to absent pharmacological activity because it may follow incorrect use, inadequate titration, or unmet expectations.

#### 3.2.3. Stage 3: Targeted Evaluation of Contributing Clinical Domains

After trial conditions and response have been characterized, evaluation should be directed by history, examination, baseline ED assessment, and whether a result would change management. Relevant domains include vascular and cardiometabolic disease; confirmed endocrine disorders; neurogenic or structural disease; medication-related contributors and prescribing safety; and psychosexual or partner-related factors [1,2,3,28,44,45,46]. These domains are non-hierarchical and frequently coexist. A contributing factor is not necessarily fully reversible, and its presence does not establish that it caused oral-treatment non-response.

Testing should be proportional to clinical indication. Early-morning fasting total testosterone is part of the core assessment for men undergoing reassessment [2]; a low result should be confirmed on a separate occasion and luteinizing hormone or prolactin obtained when the confirmed result or the clinical context warrants it [1,2]. Penile duplex Doppler ultrasonography is a conditional investigation for selected patients in whom hemodynamic information would change counseling or treatment, not a prerequisite for confirming oral non-response [2]. Duplex parameters differ across response categories in observational series, but no study has shown that duplex-guided management improves outcomes [28]. Evidence cited for PDE5-inhibitor use after nerve-sparing radical prostatectomy should not be extrapolated automatically to pelvic surgery, spinal cord injury, or all neuropathies [45,47].

Psychosexual and partner assessment should address performance anxiety, depression, sexual scripts, communication, relationship conflict, partner interest and cooperation, and sexual difficulties affecting either member of the couple. Partner-related problems are prominent among reported reasons for PDE5-inhibitor discontinuation [5]. Trials also show that successful treatment of male ED and the female partner’s sexual function and satisfaction are interrelated, supporting a dyadic assessment without assigning blame [15]. Evidence for digital psychological interventions [48] supports a treatment modality but does not validate a distinct “contextual non-response” phenotype.

#### 3.2.4. Stage 4: Shared Treatment Decision and Final Oral-Treatment Status

For this review, persistent clinically meaningful oral-PDE5-inhibitor non-response denotes continued inability to achieve an erection sufficient for the patient’s intended sexual activity after an adequately instructed and correctly administered trial of an appropriately selected PDE5 inhibitor at the maximally tolerated dose within licensed dosing, with response documented using validated outcomes when feasible. This is a descriptive oral-treatment status. It is not a stand-alone diagnosis, a diagnosis of exclusion, or proof of receptor-level or pharmacodynamic resistance.

Adequacy should be determined by principle and clinical context. No validated universal criterion requires eight attempts, trials of two agents, or mandatory once-daily tadalafil. Rechallenge, a molecule switch, or daily tadalafil may be considered when safe, accessible, tolerated, and aligned with patient preference [6,49,50,51,52,53,54,55,56], but none are required before the oral-treatment status can be recorded. Likewise, potentially contributing endocrine, cardiometabolic, medication-related, and psychosexual factors should be addressed when feasible and desired, but complete “correction” is neither always possible nor a prerequisite for second-line care.

Confirmation of oral-PDE5-inhibitor non-response must be separated from the subsequent outcome of non-oral therapy. Vacuum erection devices, intracavernosal injection, and penile-prosthesis counseling do not have to fail before oral non-response can be identified [2,29,30,31,32]. Shared decision-making may appropriately favor one of these options before every optional reassessment item has been completed, particularly in severe structural or neurogenic ED, contraindication or intolerance, cost or access barriers, or when the patient prefers a more reliable or definitive treatment.

**Table 2:** Four-stage structured reassessment framework for apparent PDE5-inhibitor non-response.

Stage and Clinical Question	Core Components	Interpretation and Next Action
1. Trial and exposure adequacy: Did the treatment have an adequate opportunity to work?	Drug, formulation, licensed source, dose, instructions, timing, relevant food and alcohol effects, sexual stimulation, number and quality of opportunities, adherence, adverse effects, cost/access, expectations, and immediate partner/contextual conditions [5,6,7,8,42,43]. Group findings as administration, pharmacokinetic exposure, adherence/supply, or behavioral/contextual barriers.	Correct remediable barriers and offer a rechallenge when safe and acceptable. These are barriers to observed effectiveness, not etiological diagnoses.
2. Observed response: What happened?	Record complete response, partial response, absent response, or discontinuation. Use IIEF-EF/SHIM, EHS, SEP2/SEP3, or GAQ when feasible and establish whether improvement is meaningful to the patient [21].	Separate outcome from cause. Analyze discontinuation independently because efficacy, tolerability, cost, inconvenience, and partner factors may differ.
3. Contributing domains: What factors may be relevant and management-changing?	Targeted assessment of psychosexual and partner factors; endocrine disorders; vascular and cardiometabolic disease; neurogenic or structural disease; and medication-related contributors and safety [1,2,3,15,28,44,45,46,47]. Morning total testosterone is part of the core assessment, consistent with guideline advice that hormonal testing include an early-morning fasting total testosterone [2]; confirmatory and second-line endocrine tests, and penile Doppler, are conditional rather than universal prerequisites.	Address relevant contributors when feasible. Domains may coexist, are not ranked by severity, and do not by themselves establish the cause of non-response.
4. Shared treatment decision and final oral status: Which option best fits the patient?	Choose between oral optimization when appropriate and established non-oral options. Re-education, maximally tolerated licensed dosing, switching, or daily tadalafil may be considered; VED, ICI, or prosthesis discussion may be selected directly according to severity, safety, tolerance, access, and preference [2,6,29,30,31,32,53,54,55,56].	Record persistent clinically meaningful oral-PDE5-inhibitor non-response after an adequately instructed and correctly administered trial with validated outcome assessment when feasible. This final oral-treatment status does not require failure of two agents, daily tadalafil, penile Doppler, correction of every contributor, or failure of non-oral treatment.

### 3.3. Residual Erectile Reserve as an Author-Proposed Physiological Hypothesis

The term residual erectile reserve is used here in a deliberately narrow sense. It denotes the author-proposed hypothesis that a patient retains a variable amount of physiological capacity across neural NO initiation, penile arterial inflow and endothelial signaling, cavernosal smooth-muscle relaxation, and veno-occlusive function [16,17,18,28]. It is not synonymous with baseline questionnaire severity, sexual desire, psychosexual context, or the observed PDE5-inhibitor response. Endocrine and psychosexual factors can influence erectile performance and are assessed separately in the structured framework rather than being used to define reserve.

An adequately observed PDE5-inhibitor response may contain indirect information about this physiological capacity, but it cannot isolate it. Dose, timing, absorption, stimulation, adherence, expectation, anxiety, partner context, and outcome measurement all affect the same observed response [5,6,7,8,21,42]. Associations between PDE5-inhibitor response and penile Doppler findings [28] do not establish a diagnostic test. No validated threshold, reference standard, test–retest reliability estimate, sensitivity or specificity, or prospectively confirmed prognostic value currently permits PDE5-inhibitor response to be called a diagnostic or prognostic “erectile reserve test.”

The 2026 Perspective by Hatzichristou presents a closely related but broader proposal, describing response as a practical “Erectile Reserve Test” and PDE5 inhibitors as initiators, enablers, and sustainers [13]. The present narrative review treats those propositions as hypothesis-generating and confines current clinical recommendations to established symptomatic facilitation and evidence-based management choices. Claims that chronic PDE5 inhibition conditions erectile tissue, prevents fibrosis, restores unassisted erections, or acts as a biological platform for other therapies are not components of the reassessment framework or of its conclusions; they are considered as questions for future research in Section 8.2. There, preclinical tissue-preservation findings are identified as preclinical [57], and improvements in drug-assisted function are distinguished from durable unassisted recovery after washout, which was not clearly established in REACTT [58,59].

## 4. Apparent Non-Response: Trial Adequacy, Treatment Selection, and Persistence

### 4.1. Inadequate Trial and Exposure Barriers

Inadequate use is one of the best-documented explanations for apparent PDE5-inhibitor non-response. In a prospective study of 250 men referred to a specialist andrology unit as non-responders, 172 (68.8%) had at least one administration error [7]. Of these 172 men, 115 accepted structured re-education, and 88 subsequently responded. The conversion rate was therefore 76.5% among the selected men who accepted re-education, but 35.2% (88/250) in the full referred cohort [7]. These denominators should not be conflated, and the findings may not generalize to unselected primary-care populations. In an earlier single-center study of 100 sildenafil non-responders, inappropriate use was identified in 56 men, and only 34 reported that a follow-up visit had been scheduled; after appropriate dose titration and timing adjustment, 31 men responded to sildenafil [8]. Together, these studies show that instruction can recover a clinically important proportion of apparent non-responders, but they do not establish that most patients initially labeled as non-responders will respond after re-education.

The adequacy of a PDE5-inhibitor trial should be assessed by principle rather than by a universal numerical rule. A reasonable clinical trial includes a licensed product obtained from a reliable source, clear instructions, a maximally tolerated licensed dose when appropriate, correct timing in relation to food and sexual activity, adequate sexual stimulation, and repeated use sufficient to judge response. Response should be documented with a validated measure where feasible; the anchor-based responder classification adopted here was validated against the International Index of Erectile Function erectile-function domain, Sexual Encounter Profile question 3, and a Global Assessment Question [21]. In one salvage study, non-response was defined as failure of at least four to six attempts at intercourse at the highest recommended dose over three months [29]; such thresholds are study eligibility criteria rather than validated diagnostic standards. Neither trials of two different agents nor once-daily tadalafil should be mandatory before a patient may be considered to have persistent non-response to oral PDE5-inhibitor therapy. A switch or daily regimen can instead be offered when clinically appropriate and acceptable to the patient.

Four types of barriers should be distinguished. Administration barriers include an inadequate dose, incorrect timing, insufficient stimulation, too few attempts, or unrealistic expectations. Pharmacokinetic exposure barriers include a high-fat meal with agents whose absorption is meal-sensitive, delayed absorption, or relevant drug interactions. Adherence and supply barriers include cost, poor persistence, counterfeit or unlicensed products, and unsupervised dose changes. Behavioral and contextual barriers include performance anxiety, avoidance, relationship difficulties, and a mismatch between the regimen and the patient’s sexual pattern. Lack of sexual stimulation is therefore not a pharmacokinetic failure. Counterfeit or online-sourced medication deserves specific attention because variable or absent active drug can create both apparent inefficacy and avoidable safety risk [43,60].

### 4.2. Selecting a PDE5 Inhibitor and Regimen

Available PDE5 inhibitors share a common mechanism but differ in half-life, food sensitivity, dosing schedule, adverse-event profile, and patient experience [24,25,26]. A 2026 network meta-analysis of 83 double-blind, placebo-controlled trials, the first to examine dose-specific outcomes, found all licensed agents efficacious against placebo and ranked sildenafil 100 mg highest, which supports dose optimization before a change of molecule is considered [61]. Network meta-analyses confirm efficacy versus placebo, although direct head-to-head evidence is more limited than the volume of placebo-controlled evidence and small comparative differences should not be overinterpreted [4,24,25]. Selection should therefore incorporate prior response, expected frequency and timing of sexual activity, adverse effects, comorbidities, concomitant medication, cost, availability, and patient preference.

Once-daily tadalafil is an established ED regimen and may suit men who prefer less treatment-linked planning, have frequent sexual activity, or have concomitant lower urinary tract symptoms associated with benign prostatic hyperplasia (LUTS/BPH) [50,51,52,62]. Tadalafil 5 mg once daily can improve both ED and LUTS/BPH in appropriately selected men [62]. This does not imply that BPH treatment in general improves or worsens erectile function. Sexual outcomes of medical and procedural BPH treatments are intervention-specific and heterogeneous [63]. Detailed comparison of BPH procedures is outside the scope of this review.

Daily tadalafil is an option, not a prerequisite for establishing persistent oral non-response. Available agents differ in pharmacokinetic profile [2] and in comparative efficacy ranking [24,25]. Switching to another PDE5 inhibitor may therefore be reasonable when duration of action, food sensitivity, tolerability, or patient preference differs. No randomized trial has evaluated switching as an intervention in men with confirmed non-response; the supporting evidence comes from uncontrolled re-education protocols in which a change of agent was combined with corrected administration, where failure to try more than one agent was the single most common error (41%) [7]. Any switch should be framed as individualized prescribing rather than as proof that one molecule is biologically superior for a particular etiological subgroup.

### 4.3. Formulation and Delivery Considerations

Alternative formulations may be useful when a practical barrier is identified, but their claims should be restricted to the evidence available. Sildenafil oral-film products have demonstrated bioequivalence to conventional film-coated tablets under fasting conditions in a single-dose pharmacokinetic study [64]. Vardenafil and sildenafil orodispersible tablets, and regionally available sildenafil films or sprays, provide alternative modes of administration [65,66,67,68]. Vardenafil orodispersible tablets are an exception to the assumption that a change of formulation is only a change of route: the product information states that the 10 mg orodispersible tablet is not bioequivalent to the 10 mg film-coated tablet, reports mean vardenafil exposure higher by 21–29% in middle-aged and elderly patients and by 44% in young healthy subjects, and caps the orodispersible dose at 10 mg daily [67]. Bioequivalence or product availability does not establish faster clinically meaningful onset, better adherence, greater acceptability, or conversion of PDE5-inhibitor non-response. Regulatory status varies by product and jurisdiction. Formulation switching should therefore be considered only when swallowing, water use, discretion, or another administration barrier is relevant; it is not a treatment for persistent non-response after an adequate oral trial.

### 4.4. Apparent Loss of Effect

Patients may report that a PDE5 inhibitor has “stopped working,” but clinically important pharmacological tolerance has not been established. The contrary position rests largely on an uncontrolled two-year survey of 82 men in which about a fifth required dose escalation and a similar proportion discontinued for diminished efficacy, from which the authors inferred a possible tachyphylaxis effect [69]; without a control group, that design cannot separate loss of drug effect from progression of the underlying vascular disease. In a randomized, double-blind study of as-needed tadalafil over six months, erectile outcomes improved and remained stable rather than showing a waning effect [70]. Tadalafil datasets extending beyond the conventional 12-week trial window are also more consistent with maintained efficacy than with tachyphylaxis: in trials of at least 24 weeks, once-daily dosing remained superior to on-demand dosing for erectile function [52]. A perceived decline should therefore prompt reassessment of administration, adherence, medication source, disease progression, new medications, hormonal status, cardiovascular or metabolic health, sexual desire, psychological state, and partner context [6,9]. Automatic dose escalation without this reassessment may increase adverse effects without correcting the cause of the change.

### 4.5. Discontinuation and Acceptability

Discontinuation is common and should not be equated with pharmacological failure. A meta-analysis of 22 studies including 162,936 patients found a mean discontinuation rate of approximately 4% per month, approaching 50% at one year; lack of efficacy and partner-related problems were among several prominent contributors [5]. Adverse effects, cost, loss of spontaneity, unrealistic expectations, inadequate follow-up, low confidence, relationship dissatisfaction, and a regimen that does not fit the couple’s sexual pattern may all contribute [2,5]. Reassessment should therefore include treatment burden, tolerability, satisfaction, and the patient’s goals, not erectile rigidity alone. Follow-up provides an opportunity to refine instructions, change a poorly matched regimen, address adverse effects, and include the partner when both individuals wish to participate.

## 5. Clinical Contributors to Persistent Poor Response

The following conditions are contributors to poor response rather than mutually exclusive “failure phenotypes.” More than one may be present, and their identification should inform management without implying that each is a separate diagnosis or that all modifiable factors must be completely corrected before another treatment is offered.

### 5.1. Diabetes, Cardiometabolic Disease, and Vasculogenic ED

Diabetes is associated with a lower probability of response because endothelial dysfunction, autonomic and peripheral neuropathy, arterial disease, impaired smooth-muscle function, and comorbidity burden may coexist [40,41]. PDE5 inhibitors nonetheless remain effective for many men with diabetic ED. Pooled effect sizes against placebo are large for sildenafil, tadalafil, and vardenafil, with numbers needed to treat of about 2.4 to 4.1 [71]. Network comparisons restricted to diabetic men find the licensed agents generally efficacious and well tolerated [72]. Erectile dysfunction is itself a marker of excess cardiovascular risk in diabetes. The randomized and observational data on PDE5-inhibitor use, by contrast, are too limited and imprecise to show any effect on cardiovascular events or mortality, and establish neither cardioprotection nor a treatment-selection benefit [73]. Severity and duration of ED, diabetes duration and control, obesity, renal function, and cardiovascular risk may help explain an individual patient’s response; they do not constitute a validated prediction score.

Penile duplex Doppler ultrasonography can characterize arterial inflow and veno-occlusive function in selected men, but it is not a routine prerequisite for confirming oral treatment non-response. It is most useful when the result is expected to alter counseling or management [2]. Neither duplex parameters nor inflammatory markers have been evaluated against a management-change endpoint [28,74]. High-sensitivity C-reactive protein was associated with response in one cohort but was not an independent predictor after adjustment and should not be used as a stand-alone biomarker [74]. A retrospective study that associated age, diabetes duration, HbA1c, obesity, and creatinine with use of dual PDE5-inhibitor regimens is hypothesis-generating; it does not validate either a treatment-selection tool or a benefit from combination therapy [75].

Cardiometabolic optimization remains important care for men with ED. Diet- and exercise-based lifestyle interventions produce a modest but significant improvement in erectile function in randomized trials [76]. Guidelines also recommend cardiovascular risk-factor modification, including smoking cessation, blood-pressure control, lipid management, and individualized glycemic care, before or alongside ED-specific treatment [2]. These measures should be recommended for their established health indications and as adjuncts to ED care, not as “metabolic restoration” or as proven methods of converting PDE5-inhibitor non-response. GLP-1 receptor agonists, dual-incretin therapies, and sodium–glucose cotransporter-2 inhibitors are not established ED rescue treatments, and current evidence does not demonstrate that they restore PDE5-inhibitor responsiveness [77,78,79,80]. One retrospective database study instead reported a higher incidence of ED among SGLT2-inhibitor users; residual confounding and indication bias limit causal interpretation, but the direction cannot support a rescue claim [81]. Their use should follow diabetes, obesity, renal, and cardiovascular indications rather than an ED-specific algorithm. Comorbidity management should not delay vacuum therapy, intracavernosal treatment, or another preferred option when oral therapy is insufficient.

### 5.2. Confirmed Hypogonadism

Low androgen status may reduce libido and contribute to ED, but biochemical and clinical criteria should be satisfied before testosterone treatment is considered. Evaluation should include symptoms and an appropriately timed morning total testosterone measurement, with confirmation of a low result on a separate occasion. Free testosterone, luteinizing hormone, prolactin, and other endocrine investigations should be obtained only when indicated by the initial result or clinical presentation [2].

A meta-analysis of eight studies in 913 hypogonadal men with ED found greater improvement with testosterone plus a PDE5 inhibitor than with PDE5-inhibitor monotherapy, with heterogeneity according to the definition of hypogonadism and prior response [44]. The TADTEST study and earlier combination studies similarly support a possible benefit in selected men with confirmed testosterone deficiency and an incomplete response to correctly used PDE5-inhibitor therapy [82,83]. These findings do not support testosterone use in eugonadal men. Treatment should follow guideline-based contraindication assessment and monitoring, and expectations should include the possibility that libido or erectile function may remain impaired despite biochemical correction [2,30,84].

### 5.3. Post-Prostatectomy and Other Neurogenic ED

Evidence after radical prostatectomy should be distinguished from evidence in other neurogenic conditions. A systematic review of 14 trials involving 2822 men after nerve-sparing radical prostatectomy found that PDE5 inhibitors improved drug-assisted erectile outcomes compared with placebo [45]. Narrative syntheses of agent-specific rehabilitation strategies reach similar conclusions but do not add pooled data [85]. This evidence should not be extrapolated to all pelvic surgery, spinal cord injury, multiple sclerosis, or peripheral neuropathy. Guideline recommendations for neurogenic erectile dysfunction are condition-specific: oral PDE5 inhibitors are recommended as a first-line medical treatment, intracavernous vasoactive injection as second-line medical treatment, and mechanical devices are offered alongside them, with some residual nerve function required for a PDE5-inhibitor response [47]. The site and completeness of the neurological injury, residual sexual function, manual dexterity, and partner involvement also bear on which of these is practicable, although the guideline does not present them as assessment criteria. Mechanistic work on cavernous-nerve injury remains hypothesis-generating rather than evidence of clinical regeneration [86].

The REACTT trial provides an important counseling distinction. Among 423 men after bilateral nerve-sparing prostatectomy, once-daily tadalafil improved drug-assisted erectile function during treatment, but unassisted erectile function after washout was not significantly better than placebo [58,59]. Daily therapy may therefore facilitate erections during use without demonstrating durable restoration of spontaneous function. A systematic review of ten studies enrolling 760 men graded the certainty of evidence for low-intensity shockwave therapy after prostatectomy as moderate for improvement in the IIEF-5 and high for safety, but low for erection hardness, for long-term functional recovery, and for combination with a PDE5 inhibitor; protocols remain unstandardized, and heterogeneity in session number, timing, and energy settings precluded meta-analysis [87]. Men with substantial nerve injury should receive realistic counseling and timely access to vacuum erection devices, intracavernosal therapy, or penile prosthesis according to goals and preference rather than being required to complete prolonged oral-therapy sequences.

### 5.4. Psychosexual, Partner, and Contextual Contributors

Contextual poor response describes a mismatch between pharmacological capacity and sexual circumstances rather than an etiological category. Erectile function may differ between masturbation, nocturnal erections, and partnered sexual activity. Performance anxiety, depression, shame, avoidance, low desire, relationship conflict, and unrealistic expectations can reduce stimulation, attention, confidence, and treatment persistence [5,6]. These factors can coexist with organic ED and should not be used to dismiss biological disease.

Partner-related factors warrant direct but non-blaming assessment. Relationship quality, the partner’s attitude toward medication, willingness to participate in treatment, sexual communication, expectations, and the practical timing of sexual activity may influence both perceived response and persistence [5,6]. Conversely, successful treatment of the man is associated with improvement in the untreated partner’s sexual function and satisfaction, supporting a dyadic view of outcome [15]. Female sexual dysfunction—including low desire, arousal or orgasmic difficulty, and genito-pelvic pain—may reduce sexual opportunity or satisfaction and can complicate assessment of the male partner’s treatment outcome [15]. It should not be presumed or treated as evidence of male drug resistance. With the partner’s consent, independent clinical evaluation may be appropriate, and couple-based goals should be agreed jointly.

Psychosexual intervention can be offered alongside medical treatment when anxiety, relationship dynamics, or inconsistent partnered response is clinically relevant. Evidence supports psychological intervention combined with PDE5-inhibitor therapy in selected men, while digital cognitive behavioral therapy and online psychoeducation may improve access but have variable adherence [48,88,89,90]. Treatment should be individualized and may include sex therapy, cognitive behavioral therapy, couple counseling, or referral for the partner’s sexual-health needs.

## 6. Safety and Tolerability During Reassessment

Safety assessment should distinguish absolute contraindications from conditions requiring cardiovascular evaluation, agent-specific dose adjustment, or cautious coadministration. All four oral PDE5 inhibitors are contraindicated with organic nitrates or nitric oxide donors, including recreational amyl or alkyl nitrite (“poppers”), and with soluble guanylate-cyclase stimulators such as riociguat; nicorandil is treated as a nitrate-related interaction [2,37,67,91,92]. If nitrate therapy may be required, PDE5-inhibitor treatment should be deferred pending cardiovascular and antianginal review [34,35]. In a life-threatening situation only, the tadalafil product information permits nitrate consideration after at least 48 h, and the avanafil product information identifies increased risk within 12 h, in both cases under close medical supervision with hemodynamic monitoring; the sildenafil and vardenafil product information provides no interval-based exception [37,67,91,92]. Patients for whom sexual activity is temporarily inadvisable because of unstable or incompletely assessed cardiovascular disease should defer sexual activity and treatment until appropriate assessment or stabilization [34,35]. Agent-specific labeled contraindications and precautions are summarized in Table 3; the current local product information should always be checked.

Alpha-blockers are not listed as an absolute class contraindication, although product information and guideline emphasis differ. Patients should be hemodynamically stable before initiation, with agent-specific starting doses and precautions: sildenafil 25 mg, vardenafil 5 mg, and avanafil 50 mg; tadalafil with doxazosin is not recommended, while the European guideline reports no general class-wide restriction on simultaneous use [2,37,67,91,92]. Controlled hypertension or use of non-alpha-blocker antihypertensive therapy does not by itself mandate deferral, but counseling about additive blood-pressure lowering and postural symptoms is appropriate [2,37,67,91,92]. Table 3 separates contraindications, reasons to defer pending assessment or stabilization, and situations requiring agent-specific dose, timing, or counseling caution.

Tolerability is clinically important because adverse effects can lead to inconsistent use, dose avoidance, or discontinuation. Common class effects include headache, flushing, dyspepsia, and nasal congestion; visual disturbance is more characteristic of sildenafil, whereas back pain and myalgia are particularly relevant to tadalafil [2,24,25,33,37]. The EAU evidence summary, adapted from regulatory product information, reports tadalafil-associated back pain in 6.5% and myalgia in 5.7%. These pooled cross-trial figures should not be extrapolated to every dose or interpreted as rates specific to tadalafil 5 mg once daily [2]. Symptoms are usually self-limited, but intolerable effects justify dose reduction, a different agent, or a non-oral option.

Counseling should address the expected onset and usual course of adverse effects and provide a plan for dose reduction, molecule or regimen change, or alternative treatment when symptoms are unacceptable. Priapism and sudden visual loss are rare but require urgent assessment under product-label recommendations [33,37,67,91,92]. Men should be told to seek immediate assistance for an erection lasting four hours or more and to stop treatment and seek immediate assessment for any sudden visual defect. For tadalafil and avanafil, the product information also instructs patients to stop treatment and seek prompt attention for a sudden decrease or loss of hearing [37,92]. Caution applies in anatomical penile deformity, including angulation, cavernosal fibrosis and Peyronie’s disease, and in conditions predisposing to priapism such as sickle cell anemia, multiple myeloma and leukemia [37,67,91,92]. Observational ocular and cardiovascular safety studies should be interpreted cautiously [93,94,95,96]. The reported association between PDE5-inhibitor exposure and lower cardiovascular-event rates does not establish causality or support prescribing these drugs for cardiovascular prevention [95,96]. Likewise, an observational association with uncommon ocular events supports risk discussion in susceptible patients but does not prove causation [93].

**Table 3:** Product-label and cardiovascular-risk classification for oral PDE5 inhibitors during reassessment. Label-derived entries are based on the current European product information for sildenafil, tadalafil, vardenafil, and avanafil [37,67,91,92]; cardiovascular-risk categories are based on the EAU guideline and Princeton IV [2,34,35]. Each condition is placed in one action column, and agent-specific differences are named. Emergency nitrate provisions are not planned coadministration pathways. The current local product information should be checked before prescribing.

Clinical Issue	Contraindicated	Defer Pending Assessment or Stabilization	Use with Agent-Specific Dose, Timing, or Counseling Caution
Nitrates and related agents	All agents: Any organic nitrate; nitric-oxide donors, including amyl or alkyl nitrite (“poppers”); riociguat. Nicorandil is treated as a nitrate-related interaction [2,37,67,91,92].	Foreseeable nitrate requirement: Defer PDE5-inhibitor treatment and review cardiovascular and antianginal management [34,35].	No planned coadministration. If nitrate is medically necessary in a life-threatening situation: Tadalafil—at least 48 h must elapse after the last dose; avanafil—use within 12 h carries increased risk. In both cases, close medical supervision and hemodynamic monitoring are required. Sildenafil and vardenafil provide no interval-based exception [37,67,91,92].
Blood pressure	All agents: Resting blood pressure below 90/50 mmHg. Tadalafil: Uncontrolled hypertension. Avanafil: Blood pressure above 170/100 mmHg [37,67,91,92].	Symptomatic orthostasis, hemodynamic instability, or uncertain blood-pressure control: Assess and stabilize before first exposure [34,35].	Controlled hypertension or non-alpha-blocker antihypertensive therapy is generally compatible with treatment; counsel regarding additive blood-pressure lowering and apply the alpha-blocker precautions below [37,67,91,92].
Recent cardiovascular events	Sildenafil: Recent myocardial infarction or stroke, without a numerical interval in the product information. Tadalafil: Myocardial infarction within 90 days or stroke within 6 months. Vardenafil: Myocardial infarction or stroke within 6 months. Avanafil: Myocardial infarction, stroke, or life-threatening arrhythmia within 6 months [37,67,91,92].	An event outside the labeled interval, incomplete revascularization, new or worsening symptoms, or a recent change in cardiac status: Defer until cardiovascular risk stratification is complete [34,35].	Stable, low-risk coronary disease after assessment: Eligible for treatment; no label-mandated dose or timing modification applies solely because coronary disease is stable [34,35].
Cardiac status and exercise tolerance	Sildenafil: Severe cardiovascular disease in which sexual activity is inadvisable, including unstable angina or severe cardiac failure. Tadalafil: Unstable angina or angina during sex, NYHA class II or greater heart failure within 6 months, or uncontrolled arrhythmia. Vardenafil: Unstable angina or severe cardiac failure (NYHA III/IV). Avanafil: Unstable angina, angina during sex, or NYHA class II or greater heart failure [37,67,91,92].	Any patient for whom sexual activity is currently inadvisable, and patients with indeterminate or intermediate cardiovascular risk, uncertain exercise tolerance, or symptoms with low-level exertion: Defer until assessment or stabilization is complete [34,35].	Low-risk, well-compensated cardiovascular disease: Treatment may proceed after routine assessment; counsel regarding symptoms during sexual activity [34,35].
Ophthalmic	Prior NAION-related loss of vision in one eye: All agents. Known hereditary degenerative retinal disorders: Sildenafil, vardenafil, and avanafil; not stated as a tadalafil contraindication [37,67,91,92].	Unresolved visual symptoms: Defer until appropriately assessed. This is a prudent clinical action, not an additional labeled contraindication [37,67,91,92].	All agents: Stop treatment and seek immediate medical assessment for any sudden visual defect [37,67,91,92].
Hepatic and renal function	Sildenafil: Severe hepatic impairment. Vardenafil: Child–Pugh C or end-stage renal disease requiring dialysis. Avanafil: Child–Pugh C or creatinine clearance below 30 mL/min. Severe hepatic or renal impairment is not a labeled tadalafil contraindication [37,67,91,92].	—	Sildenafil: Consider 25 mg in hepatic impairment or severe renal impairment; usual dosing applies in mild–moderate renal impairment. Tadalafil: No adjustment in mild–moderate renal impairment; severe renal impairment—maximum 10 mg on demand and no once-daily dosing; severe hepatic impairment requires individual benefit–risk assessment. Vardenafil: Start 5 mg in Child–Pugh A/B (maximum 10 mg in B) or severe renal impairment; no adjustment in mild–moderate renal impairment. Avanafil: No adjustment in mild–moderate renal impairment; start the minimum effective dose in Child–Pugh A/B [37,67,91,92].
Interacting medication	Avanafil: Potent CYP3A4 inhibitors. Vardenafil: ritonavir or indinavir at any age; oral ketoconazole or itraconazole in men older than 75 years [67,92].	—	Sildenafil: Consider 25 mg with CYP3A4 inhibitors; ritonavir is not advised and, if coadministered, sildenafil must not exceed 25 mg/48 h. Tadalafil: Use potent CYP3A4 inhibitors cautiously because exposure increases. Vardenafil: With erythromycin or clarithromycin, do not exceed 5 mg; avoid grapefruit; QT-prolonging drugs are best avoided in patients with relevant risk factors. Avanafil: With moderate CYP3A4 inhibitors, maximum 100 mg/48 h; avoid grapefruit juice for 24 h before dosing [37,67,91,92].
Alpha-blockers	—	Sildenafil, vardenafil, and avanafil: Hemodynamic instability on alpha-blocker therapy—stabilize before initiation. For tadalafil, review symptomatic hypotension risk, especially with doxazosin [37,67,91,92].	Sildenafil: Consider 25 mg; symptomatic hypotension is most likely within 4 h after dosing. Vardenafil: Start 5 mg; it may be given at any time with tamsulosin or alfuzosin, while dose separation should be considered with other alpha-blockers. Avanafil: Start 50 mg. Tadalafil: Use other alpha-blockers cautiously with minimal-dose initiation and progressive adjustment; doxazosin combination is not recommended. The EAU reports no general class-wide restriction on simultaneous use, but agent-specific label restrictions prevail [2,37,67,91,92].
Priapism risk and penile anatomy	—	—	All agents: Use cautiously with penile angulation, cavernosal fibrosis, Peyronie’s disease, sickle-cell anemia, multiple myeloma, or leukemia. Counsel that an erection lasting 4 h or more requires immediate medical assistance [2,37,67,91,92].
Hearing	—	—	Counsel all patients to stop treatment and seek prompt medical attention for a sudden decrease or loss of hearing. This instruction is explicit in the tadalafil and avanafil product information and is retained here as class counseling; the sildenafil and vardenafil product information lists hearing-loss events without the same Section 4.4 instruction [2,33,37,67,91,92].

## 7. Structured Reassessment and Shared Decision-Making

**Figure 2:**
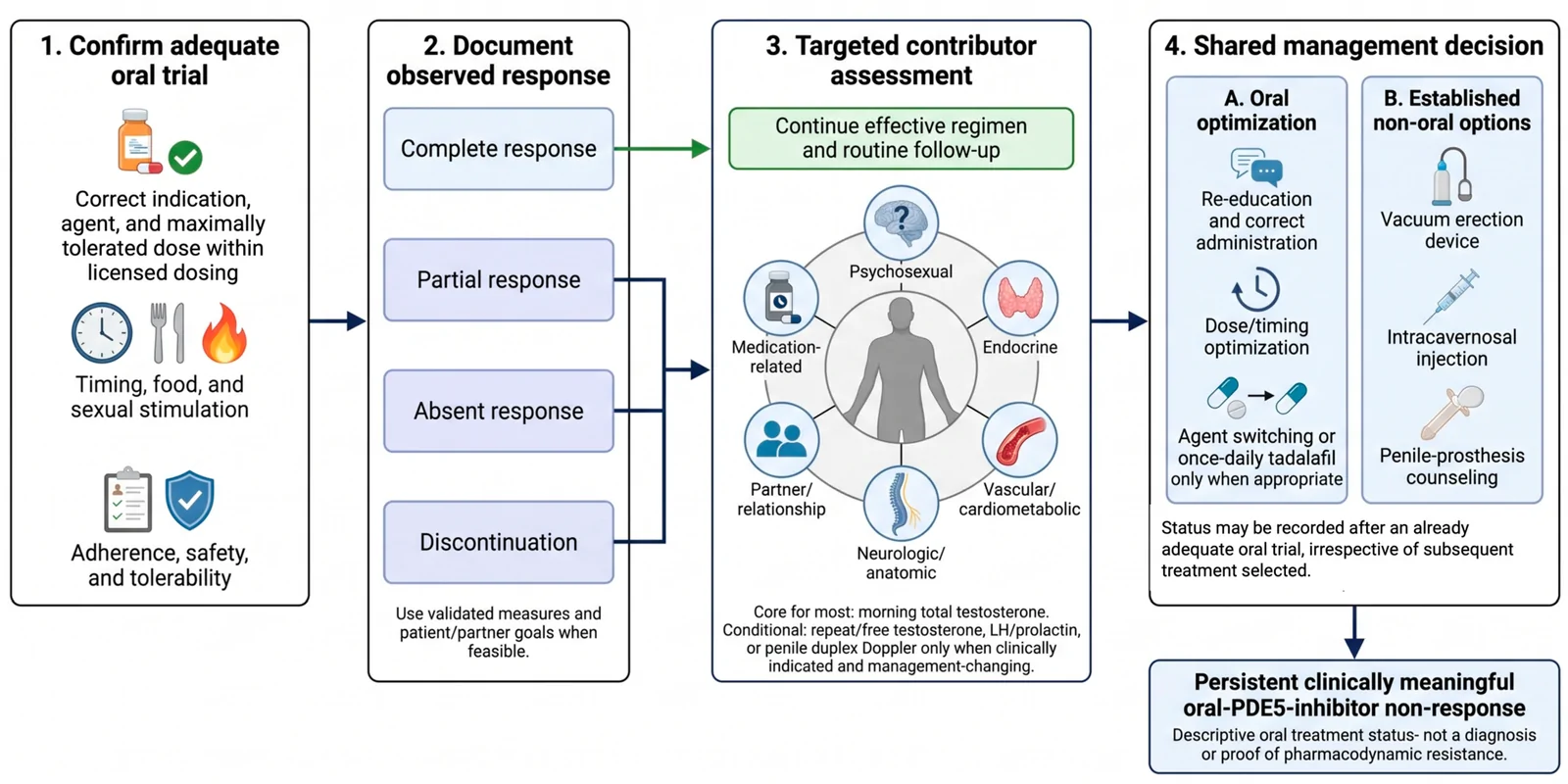
Structured clinical reassessment of apparent oral PDE5-inhibitor non-response: an author-proposed, four-step shared decision-making pathway. Core reassessment confirms treatment exposure, safety, tolerability, goals, and response using validated measures where feasible. An adequate oral trial uses the maximally tolerated dose within licensed dosing. Targeted evaluation is then limited to contributing domains suggested by the initial assessment. Oral optimization and established non-oral options are discussed in parallel through shared decision-making. Switching agents, once-daily tadalafil, and penile duplex Doppler appear only in conditional branches. Established non-oral options are shown as selectable at this decision point rather than only after oral optimization has failed, according to contraindications, tolerability, anatomy, access, or patient preference. The final oral-treatment status may be recorded after an already adequate oral trial irrespective of the subsequent treatment selected. Persistent clinically meaningful oral-PDE5-inhibitor non-response is shown as a final clinical status rather than an etiological phenotype. The pathway is an exploratory educational synthesis, not a validated diagnostic instrument or mandatory sequence. Created in BioRender. Kaltsas, A. (2026). https://BioRender.com/t7mmhiz.

The approach below is an author-proposed educational synthesis of established assessment elements and treatment options, not a validated classification, diagnostic instrument, or mandatory algorithm. It overlaps with earlier non-responder management reviews and with contemporary 2026 syntheses [6,9,12,13,49]. Its purpose is to organize reassessment while preserving clinical judgment and patient choice. Three complementary views of the same process are used: Table 2 gives the four stages, which describe what question is being answered; Table 4 gives three tiers, which describe how intensively to investigate and map onto Stages 1 to 3; and Figure 2 gives four steps, which show the order of the clinical encounter. The stages describe purpose, the tiers describe intensity, and the steps describe sequence; they are not three different frameworks.

### 7.1. Three Tiers of Proposed Reassessment Domains

Reassessment can be divided into three tiers. Tier 1 contains core information appropriate for most patients. Tier 2 contains targeted tests or assessments triggered by the history, examination, or likely effect on management. Tier 3 contains specialist or exploratory investigations that should not be routine. The tiers describe the intensity of assessment, not stages of biological disease, and are set out in Table 4.

This table is a proportional decision aid. It should not be interpreted as a requirement to complete every item before offering a vacuum device, intracavernosal injection, or penile prosthesis. Shared decision-making is particularly important in severe structural or neurogenic ED, after major pelvic surgery, when oral treatment is poorly tolerated, or when the patient prefers a faster or more definitive option [30,35].

### 7.2. Management Options After Reassessment

Management should match the correctable barrier, contributing condition, degree of response, safety profile, and patient preference. Oral optimization and established non-oral options should be discussed in parallel rather than as an inflexible ladder. Table 5 sets out the options considered below, with the evidence context, guideline position, and regulatory status of each reported separately.

#### 7.2.1. Re-Education and Adequately Instructed Rechallenge

Re-education is appropriate when the first trial was inadequately instructed or administered. It should cover licensed dose, timing, food effects, sexual stimulation, expected onset and duration, repeated use sufficient to assess response, adverse effects, and partner communication where appropriate [6,7,8]. In the 250-man referral cohort, 88 of 115 men who accepted re-education responded (76.5%), but these 88 represented 35.2% of the original cohort [7]. A separate real-world study of 100 consecutive tadalafil non-responders and 100 consecutive vardenafil non-responders reported conversion in 52% and 46%, respectively, after sequential re-instruction and regimen optimization [53]. These studies support a targeted rechallenge when an inadequacy is identified; they do not justify the statement that most referred non-responders are recovered by education.

#### 7.2.2. Dose, Molecule, and Regimen Optimization

If treatment was tolerated but underdosed, titration to the maximally tolerated licensed dose is appropriate [6]. Switching agents can be considered when duration, meal sensitivity, onset expectations, or adverse effects make the first agent a poor fit, although randomized evidence for switching confirmed non-responders is limited [24,25]. Once-daily tadalafil may be considered for men who prefer a daily regimen, have frequent sexual activity or LUTS/BPH, experience anxiety around planned dosing, or have had an unsatisfactory on-demand trial [51,52,54,55]. Continuation and satisfaction with once-daily tadalafil are high irrespective of comorbidity, but observed erectile-function gains are smaller in men with benign prostatic hyperplasia and in those previously treated with a PDE5 inhibitor, so these features indicate a possible regimen preference rather than a predictor of greater benefit [56]. It is not mandatory, and contraindication, intolerance, cost, availability, and patient preference may make it inappropriate. An alternative formulation is relevant only when a specific administration barrier exists and should not be represented as biological rescue [64,66,67,68,99].

#### 7.2.3. Specialist-Supervised Oral Combination Therapy

Combining once-daily tadalafil with an on-demand short-acting PDE5 inhibitor increases cumulative exposure and remains off-label. It should be considered, if at all, only after careful cardiovascular and interaction review in specialist practice. One randomized study in a general ED population reported greater improvement with tadalafil plus sildenafil than with tadalafil alone, particularly among men with severe ED, but it did not validate selection based on diabetes or establish efficacy in all monotherapy-resistant patients [100]. A systematic review restricted to men in whom PDE5-inhibitor monotherapy had failed found no advantage for adding an androgen or an alpha1-adrenoceptor antagonist, judged combinations with metformin, folic acid, or a 5-alpha-reductase inhibitor uncertain, and concluded that an unmet need for oral treatment in non-responders remains [101]. A systematic review and meta-analysis of combination therapies versus monotherapy suggests possible benefit in broader populations, but the evidence is heterogeneous, and several subgroup estimates rely on small or single studies [102]. This approach is not a standard prerequisite before established second-line treatment and should not be presented as a multimodal “platform” strategy.

#### 7.2.4. Testosterone Treatment in Confirmed Hypogonadism

Testosterone treatment may be considered for men with compatible symptoms and confirmed biochemical hypogonadism who have an incomplete response to correctly used PDE5-inhibitor therapy [44,83]. In the only dedicated randomized trial, the add-on effect of testosterone was confined to men with a baseline total testosterone of 3 ng/mL or below; the overall result was null [82]. It should not be used in eugonadal men or as a substitute for an adequate oral trial. The decision should follow current hypogonadism guidance, including repeat biochemical confirmation, contraindication assessment, discussion of fertility, and ongoing clinical and laboratory monitoring [2]. The goal is treatment of testosterone deficiency; improved PDE5-inhibitor response is a possible secondary benefit rather than a guaranteed outcome.

#### 7.2.5. Vacuum Erection Devices

A vacuum erection device (VED) is an established non-oral option that can be offered alone or with a PDE5 inhibitor. In a small study of 69 men with prior PDE5-inhibitor non-response, combined VED and PDE5-inhibitor treatment improved IIEF-5 and intercourse outcomes [29]. A subsequent systematic review and meta-analysis supports effectiveness across several difficult-to-treat populations, although study designs and definitions were heterogeneous [31]. Bruising, discomfort, numbness, and dissatisfaction with the mechanical nature of treatment should be discussed. VED can be offered without requiring further oral trials, particularly when the patient prefers a non-invasive, drug-sparing option.

#### 7.2.6. Non-Oral Pharmacological Therapy and Penile Prosthesis

Alprostadil can also be given without a needle. Both guidelines cited here recommend that men be informed about intraurethral alprostadil, and the European guideline additionally describes topical alprostadil with a permeation enhancer; erections sufficient for intercourse are reported in roughly a third to two thirds of men, with local pain the commonest adverse effect and fibrosis and priapism rare [2,30]. These options matter here because they suit precisely the man who wants something less invasive than injection, and omitting them would narrow choice while claiming to widen it. Intracavernosal vasoactive therapy is an established non-oral treatment for men with an inadequate oral response [103], and a proportion of men who do respond to oral therapy still prefer injection for its reliability [104]. It need not be reached through a fixed sequence: the American Urological Association states that all treatment modalities that are not contraindicated should be presented as potential first-line options, regardless of invasiveness or irreversibility, within shared decision-making [30]. Training, dose titration, priapism precautions, penile pain, and fibrosis risk should be addressed. A penile prosthesis is an established definitive option for persistent ED when less invasive treatment is ineffective, unacceptable, or not preferred, and it is associated with high device reliability and patient satisfaction in appropriately counseled men [30,32]. Neither option should be delayed solely to complete an author-proposed sequence of multiple oral agents, daily tadalafil, Doppler testing, or every potentially modifiable comorbidity.

#### 7.2.7. Low-Intensity Shockwave Therapy

Low-intensity shockwave therapy (LiSWT) may be discussed with selected, well-informed men with vasculogenic ED, but its evidence and regulatory position differ from those of VED, intracavernosal therapy, and prosthesis. A double-blind, sham-controlled study reported conversion of some PDE5-inhibitor non-responders [105]. By contrast, a general-ED meta-analysis does not directly establish conversion among non-responders [106], and a recent Cochrane review judged the evidence low certainty, with heterogeneous protocols and uncertain clinical importance [107]. In severe ED, a sham-controlled trial evaluated LiSWT added to daily tadalafil [108]. In mild or mild-to-moderate ED, a separate randomized study compared LiSWT plus tadalafil with LiSWT plus placebo, so its result describes the effect of adding tadalafil to LiSWT rather than the effect of adding LiSWT to tadalafil [109]. LiSWT should remain an expectation-managed, low-certainty option, with guideline and device-regulatory status verified locally.

#### 7.2.8. Psychosexual and Couple-Based Care

Psychosexual treatment may be offered alongside medical therapy when performance anxiety, low confidence, inconsistent contextual response, relationship distress, or partner-related barriers are present. Digital cognitive behavioral therapy and psychoeducation can improve access, although adherence and long-term evidence remain limitations [48,88,89]. A review and meta-analysis supports combining psychological intervention with PDE5-inhibitor therapy in selected men [90]. Couple-based care should address communication, expectations, sexual scripts, and the needs of both partners; partner sexual dysfunction should prompt an appropriate independent evaluation when desired [15]. These interventions are adjunctive, preference-sensitive care and should not be used to imply that persistent poor response is primarily psychological.

**Table 5:** Management options after an unsatisfactory PDE5-inhibitor response. Evidence context, guideline position, and regulatory status are reported separately; this table is not a formal certainty-of-evidence assessment.

Strategy	Direct Evidence in Prior Non-Responders	Guideline Position	Regulatory/Off-Label Status	Practical Use and Key Evidence
Correct instruction and rechallenge	Prospective referral and salvage cohorts; denominator and selection limitations apply	Standard first corrective step when use was inadequate	Not applicable; use licensed product and dose	Identified administration, exposure, adherence, or expectation barrier. Key evidence: [6,7,8,53].
Licensed-dose titration or molecule/regimen change	Direct switching evidence is limited; daily tadalafil has selected non-responder data	Accepted oral optimization; not mandatory before second-line treatment	Each drug/dose must follow local label; switching is not a separate indication	Underdosing, partial response, food/timing mismatch, adverse effects, or preference mismatch. Key evidence: [6,24,25,51,52,54].
Once-daily tadalafil	Selected studies in prior on-demand non-responders; not a universal prerequisite	Established option when clinically appropriate	Approved for ED, and for LUTS/BPH in many jurisdictions; verify local label	Preference for daily readiness, frequent activity, LUTS/BPH, or selected unsatisfactory on-demand response. Key evidence: [50,51,52,54,55,56,62].
Dual PDE5-inhibitor therapy	Limited; studies include general and heterogeneous resistant populations	Not standard care; evidence remains limited	Off-label; additive hypotension and adverse effects require counseling	Highly selected persistent partial response under specialist supervision. Key evidence: [100,101,102].
Testosterone plus PDE5 inhibitor	Meta-analysis and selected hypogonadal non-responder studies	Treat confirmed testosterone deficiency, not eugonadal ED	Testosterone approved for indicated hypogonadism; add-on combination not separately licensed	Symptoms and repeatedly confirmed biochemical hypogonadism with incomplete response. Key evidence: [44,82,83,84].
VED alone or with a PDE5 inhibitor	Small direct non-responder study plus heterogeneous meta-analysis	Established non-oral option	Use a locally authorized device; the combination is not a separate indication	Preference for non-invasive mechanical therapy; persistent ED after inadequate oral response, including after radical prostatectomy. Key evidence: [29,30,31].
Intraurethral or topical alprostadil	No dedicated non-responder trial; efficacy data come from broader ED populations	Recommended for discussion by both guidelines; weak recommendation in the European guideline	Licensed products and formulations vary by jurisdiction	Preference for a non-oral option without injection; needle aversion; erections sufficient for intercourse in about a third to two thirds of men, with local pain the commonest adverse effect. Key evidence: [2,30].
Intracavernosal therapy	Evidence largely derives from broader ED and difficult-to-treat populations	Established non-oral option, not confined to a fixed second-line position	Approved agents and formulations vary by jurisdiction	Inadequate oral response or preference for a more reliable non-oral treatment. Key evidence: [30,103,104].
Penile prosthesis	Not dependent on proving pharmacological resistance	Established definitive option, not confined to salvage use	Regulated device; model and indication vary by jurisdiction	Persistent ED with failure, intolerance, or rejection of less invasive treatments; preference for definitive therapy. Key evidence: [30,32].
LiSWT	One sham-controlled non-responder study; broader evidence is heterogeneous and low certainty	Guideline recommendations are weak or variable	ED-specific device approval varies and is absent in some jurisdictions	Selected, well-informed men with vasculogenic ED. Key evidence: [105,106,107,108,109,110].
Psychosexual, digital, or couple-based intervention	Mixed ED populations; digital evidence does not validate a distinct phenotype	Appropriate adjunct when clinically indicated	Not applicable	Anxiety, contextual variability, relationship or partner factors, or partial pharmacological response. Key evidence: [15,48,88,89,90].

Formulation switching and cardiometabolic optimization are not listed as rescue strategies. Alternative formulations may address an administration barrier, while cardiometabolic care should follow its own clinical indications. Neither currently has direct evidence of converting persistent oral PDE5-inhibitor non-response.

### 7.3. Author-Proposed Shared-Decision Pathway

The pathway begins with a core reassessment of diagnosis, severity, treatment exposure, validated response, safety, tolerability, and goals. A clinically meaningful response should be assessed with validated patient-reported or functional outcomes where feasible [21]. The next step is targeted evaluation of contributing domains—endocrine, vascular, neurological or postsurgical, medication-related, psychosexual, and partner-related—only when suggested by the core assessment and when the result can change management.

Management is then selected through shared decision-making. When an administration or exposure barrier is present, corrected instruction and an adequately administered rechallenge are reasonable. Dose titration, a molecule switch, or once-daily tadalafil may be chosen when appropriate, but none are a universal prerequisite. At the same decision point, VED, intracavernosal therapy, and penile prosthesis should be discussed according to severity, prior experience, expected reliability, invasiveness, adverse effects, cost, access, and patient preference [30,35]. Selected adjuncts such as testosterone for confirmed hypogonadism, psychosexual or couple-based care, and expectation-managed LiSWT should be linked to their specific indications and evidence limitations.

Persistent clinically meaningful oral-PDE5-inhibitor non-response is defined here exactly as in Section 3.2.4: continued inability to achieve an erection sufficient for the patient’s intended sexual activity after an adequately instructed and correctly administered trial of an appropriately selected PDE5 inhibitor at the maximally tolerated dose within licensed dosing. A clinically meaningful but incomplete improvement is a partial response and is recorded as such, not as non-response. It is a clinical status, not a stand-alone diagnosis or etiological phenotype. It does not require a fixed number of attempts, trials of two agents, mandatory daily tadalafil, universal penile Doppler, complete correction of every comorbidity, or failure of non-oral treatment. The pathway is shown in Figure 2. A patient may appropriately choose an established non-oral or definitive option earlier, particularly in severe structural or neurogenic ED, after major pelvic surgery, when adverse effects preclude oral optimization, or when treatment preference favors reliability over further oral experimentation.

### 7.4. A Contemporaneous Proposal, and Where This Review Differs

A 2026 Perspective proposes that the response to PDE5 inhibitors be interpreted as a practical “Erectile Reserve Test,” and suggests that a short trial of daily PDE5 inhibition, typically 5 mg tadalafil once daily for approximately two weeks, allows for a more reliable assessment than on-demand dosing because steady-state levels are reached, and multiple attempts can occur [13]. The pharmacokinetic rationale is sound, and the criticism of single on-demand attempts is one this review shares: timing, food effects and an insufficient number of attempts are precisely the exposure variables that Stage 1 of the present framework examines. This review nonetheless does not adopt the proposal as a diagnostic standard, for four reasons. First, no diagnostic-accuracy study has evaluated the two-week protocol against any reference standard; the proposal is advanced in explicitly conditional terms and is not accompanied by sensitivity, specificity or predictive-value data [13]. Second, the three response patterns it describes are qualitative, without a threshold on any validated instrument, so classification is not reproducible between clinicians. Third, the proposal itself notes that an absent response may reflect performance anxiety or increased sympathetic tone rather than organic disease [13]; a test that does not separate advanced vasculogenic disease from psychogenic non-response is not performing the discrimination that would justify a diagnostic label. Fourth, were a two-week daily trial to become a prerequisite, it would delay discussion of vacuum therapy, intracavernosal injection or prosthesis in men who have already completed an adequate on-demand trial, a delay that neither the European guideline nor the international consultation recommendations require [1,2]. Daily tadalafil therefore remains, in this review, a therapeutic option to be selected by shared decision-making rather than a diagnostic step to be completed. “Residual erectile reserve” is retained here only as a physiological construct; establishing it as a test would require prospective evaluation against hemodynamic or outcome reference standards, with predefined response thresholds.

## 8. Limitations and Future Research

### 8.1. Limitations

This narrative review did not use a preregistered systematic-review protocol, formal risk-of-bias assessment, or GRADE process. Its search and selection methods are therefore reported in full in Section 2 and Supplementary Method S1, and selection and citation bias cannot be excluded. The evidence base includes guidelines, reviews, randomized and prospective studies, retrospective cohorts, preclinical work, product information, and regulatory or manufacturer sources used for limited purposes. These source types cannot support equivalent levels of inference.

The structured reassessment approach is an author-generated synthesis. It was not developed through external expert consensus, Delphi methods, patient or partner input, content-validity testing, reliability assessment, or prospective outcome evaluation. Table 6 sets out, source by source, what this review adds to that literature and what it does not. Its components overlap substantially with earlier non-responder management publications and with contemporary 2026 reviews and perspectives [6,9,12,13,49]. Citations supporting an individual assessment or treatment do not validate the framework as a whole.

Several clinical thresholds and proposed constructs remain unvalidated. In particular, no universal evidence requires eight attempts, two different PDE5 inhibitors, mandatory daily tadalafil, or completion of all reversible-factor treatment before persistent oral non-response can be described. “Residual erectile reserve” is likewise used throughout as an author-proposed physiological hypothesis rather than as a diagnostic or prognostic test (Section 3.3). PDE5-inhibitor response is influenced by dose, timing, stimulation, adherence, expectations, partner context, and pharmacokinetics and cannot currently serve as a specific measure of biological reserve.

The literature is heterogeneous in population, baseline severity, definition of non-response, outcome measure, and follow-up. Some claims are supported by studies in general ED populations rather than confirmed PDE5-inhibitor non-responders. Regulatory status also varies across jurisdictions and changes over time. Table 5 therefore separates direct non-responder evidence, guideline position, and regulatory status rather than assigning informal evidence-certainty labels.

### 8.2. Future Research Priorities

Prospective studies should establish a reproducible definition of an adequately administered PDE5-inhibitor trial and use validated outcomes to distinguish absent response, partial response, discontinuation, and patient preference. Any proposed reassessment framework should undergo patient and partner input, content-validity assessment, inter-rater reliability testing, prospective validation, and evaluation against patient-important outcomes. Comparative work should determine whether the framework adds value beyond established guideline-based care and recently published management syntheses [12,111].

Claims of vascular or tissue conditioning require separate testing from acute drug-assisted erection. Human studies support selected endothelial-function and drug-assisted outcomes, while historical rehabilitation concepts remain unvalidated as disease modification [112,113]. Trials should distinguish erectile function during treatment from durable unassisted function after washout, as illustrated by the REACTT findings [58,59]. Mechanistic and animal evidence, including the preclinical study of chronic tadalafil and corporal fibrosis [57], should generate hypotheses rather than clinical claims. The overlapping 2026 Perspective proposing PDE5 inhibitors as initiators, enablers, and sustainers should be discussed as a conceptual model, not as validation of diagnostic testing or disease modification [13].

Cardiometabolic trials should test whether weight loss, exercise, or contemporary diabetes and obesity treatments alter PDE5-inhibitor responsiveness as a prespecified outcome rather than inferring conversion from general erectile-function improvement. Studies of oral combinations, LiSWT, and other adjuncts should use clearly defined prior non-response, adequate comparators, standardized protocols, and durability outcomes. Partner satisfaction, partner sexual function, adherence, adverse effects, and discontinuation should be included alongside erectile-function scores.

Intracavernosal botulinum toxin has been tested in two randomized trials conducted specifically in men refractory to PDE5 inhibitors, with improvement in erectile-function scores against placebo, but the European guideline concludes that no recommendation for its use in clinical practice can be made, and it should be regarded as investigational rather than as an option to offer [2]. Regenerative approaches remain investigational. A review of autologous immune-cell-based strategies proposes that modulation of macrophage activity, angiogenesis, endothelial repair, and tissue regeneration could be relevant to vasculogenic ED, but it does not provide clinical evidence for treating PDE5-inhibitor non-response [114]. This immunocentric hypothesis, stem-cell approaches, and platelet-rich plasma should remain confined to well-designed clinical research; a recent synthesis found no convincing superiority of platelet-rich therapies over placebo [115]. Gut-microbiome hypotheses, nanomedicine-enabled approaches, and alternative pharmacological targets are likewise research topics rather than current rescue options [116,117,118]. These emerging topics should not displace established assessment or treatment options in the present clinical pathway.

## 9. Conclusions

An unsatisfactory response to a PDE5 inhibitor is a clinical starting point rather than a single diagnosis. Reassessment should confirm that the patient received an adequately instructed, correctly administered, tolerated trial and should document response with validated measures where feasible. Administration and exposure barriers, partial response, endocrine or cardiometabolic disease, vascular or neurological injury, medication effects, and psychosexual or partner-related factors may coexist. Correcting an identifiable barrier can recover response in some patients, but the available referral data do not show that most initially labeled non-responders will convert after re-education.

Persistent clinically meaningful oral-PDE5-inhibitor non-response is best used as a descriptive clinical status after an adequate oral trial, not as proof of pharmacodynamic resistance or as a stand-alone diagnosis. No universal evidence mandates eight attempts, two agents, daily tadalafil, penile Doppler, or completion of every proposed reassessment item. Oral optimization and established non-oral treatments should be considered through shared decision-making, and severe structural or neurogenic disease, adverse effects, or patient preference may appropriately lead to VED, intracavernosal therapy, or penile prosthesis without prolonged oral sequencing. The proposed reassessment pathway is an updated, pragmatic, author-proposed educational synthesis that requires prospective evaluation before claims of diagnostic, prognostic, or outcome benefit can be made.

## Figures and Tables

**Table 1:** Common misconceptions and evidence-aligned clinical implications in PDE5-inhibitor therapy.

Common Misconception	Evidence-Aligned Interpretation	Clinical Implication
“The pill creates an erection.”	PDE5 inhibition amplifies NO–cGMP signaling; it does not initiate sexual arousal or neural NO release [16,17,18,19,20].	Explain the need for stimulation and the expected onset and duration before judging response.
“One unsuccessful use proves treatment failure.”	Response depends on adequate instruction, licensed dosing, timing, exposure, and a meaningful opportunity to assess effect [6,7,8]. No universal fixed number of attempts has been validated.	Review the trial and offer a correctly administered rechallenge when acceptable; do not impose an arbitrary attempt threshold.
“Apparent non-response is one diagnosis.”	Trial adequacy, observed outcome, contributing domains, and final oral-treatment status are different conceptual axes [6,9,10,21].	Use the four-stage structured reassessment framework; do not assign an etiological label from the outcome alone.
“All PDE5 inhibitors are interchangeable.”	Agents share a mechanism but differ in dosing schedule, pharmacokinetics, food effects, adverse-effect profile, and fit with sexual preferences [22,23,24,25,26,27].	Select the regimen collaboratively; switching or daily tadalafil is optional, not a diagnostic prerequisite.
“No response proves pharmacodynamic resistance.”	Apparent non-response can reflect limited exposure or contextual factors, while persistent response failure may reflect severe vascular, neural, smooth-muscle, or structural impairment; no validated resistance test exists [6,7,8,10,28].	Reassess first, measure the outcome, and avoid language implying proven molecular resistance.
“All reversible factors must be corrected before second-line treatment.”	Contributing conditions may be only partly modifiable, and treatment priorities differ among patients [1,2,3].	Address relevant factors when feasible, but use shared decision-making and do not delay a preferred established therapy.
“Failure of an oral drug means that penile prosthesis is the only next option.”	Vacuum erection devices and intracavernosal injection are established non-oral options; prosthesis counseling is appropriate when desired or indicated [2,29,30,31,32].	Discuss effectiveness, burden, adverse effects, cost, and patient preference across established options.
“PDE5 inhibitors are broadly cardiotoxic,” or “all alpha-blocker use is absolutely contraindicated.”	Nitrates/NO donors and riociguat are absolute contraindications under current jurisdiction-specific labeling. Resting hypotension below 90/50 mmHg is a labeled contraindication for all four oral PDE5 inhibitors. Unstable cardiovascular disease may require stabilization before sexual activity; borderline or labile blood pressure, selected alpha-blocker use, and relevant interactions require individualized assessment and dose/timing management [2,33,34,35,36,37].	Separate absolute contraindications from conditions requiring cardiovascular assessment or cautious prescribing.
“Treatment response concerns only the man.”	Adherence, opportunity, satisfaction, and discontinuation can be influenced by relationship context and sexual difficulties affecting either partner [5,15].	Ask about partner context and female sexual difficulties respectfully and offer couple-based assessment when appropriate.

**Table 4:** Proposed reassessment domains after an unsatisfactory PDE5-inhibitor response.

Tier	Domain	Assessment	Purpose and Limits
1. Core for most patients	Treatment exposure	Agent, formulation, licensed source, dose, timing, food and alcohol, stimulation, adherence, number and circumstances of attempts	Identifies inadequate instruction, administration, exposure, adherence, or supply barriers; no fixed number of attempts is universally diagnostic.
1. Core for most patients	Validated response assessment	IIEF-EF or SHIM; EHS; SEP2/SEP3; GAQ where feasible	Distinguishes absent, partial, and satisfactory response using patient-reported outcomes rather than the statement that “the pill failed.”
1. Core for most patients	Clinical reassessment	ED onset and course; morning/spontaneous erections; libido; ejaculation and orgasm; penile pain or curvature; medical, surgical, neurological, and medication history; focused examination	Detects comorbid, iatrogenic, structural, endocrine, or neurological contributors.
1. Core for most patients	Cardiovascular safety	Cardiovascular symptoms and exercise tolerance; nitrate/NO-donor or riociguat use; blood pressure; relevant drug interactions	Identifies absolute contraindications and patients needing cardiovascular evaluation; alpha-blocker use generally requires caution, not automatic exclusion.
1. Core for most patients	Tolerability and goals	Adverse effects, treatment burden, cost, spontaneity, preferred route, willingness to continue oral therapy, and interest in established alternatives	Prevents repeated prescribing of an unacceptable regimen and supports shared decisions.
1. Core for most patients	Psychosexual and partner context	Anxiety, mood, desire, solitary versus partnered response, communication, relationship factors, and partner participation when mutually desired	Identifies contextual barriers without assigning blame or treating them as proof of psychogenic ED.
1. Core for most patients	Basic risk assessment	Blood pressure, BMI/waist as appropriate, smoking; glucose or HbA1c, lipids, and morning total testosterone according to guideline-based ED evaluation	Supports cardiovascular, metabolic, and endocrine care; these measures do not predict PDE5-inhibitor response with validated accuracy.
2. Conditional, based on indication	Endocrine clarification	Repeat low testosterone; free testosterone where indicated; LH and prolactin after confirmed low testosterone or specific symptoms	Avoids routine indiscriminate hormone panels and confirms clinically relevant hypogonadism.
2. Conditional, based on indication	Organ function and interactions	Renal/hepatic function, medication interaction review, and dose adjustment	Applied when comorbidity, age, or concomitant medication changes exposure or safety.
2. Conditional, based on indication	Vascular assessment	Penile duplex Doppler ultrasonography	Reserved for selected men when the result will alter counseling or management; not a prerequisite for defining persistent oral non-response.
2. Conditional, based on indication	Focused psychosexual/couple assessment	Validated mood or sexual-function measures; sex-therapy or couple assessment; partner evaluation with consent	Used when anxiety, relationship dynamics, variable contextual response, or partner sexual dysfunction is clinically relevant.
2. Conditional, based on indication	Neurological or postsurgical assessment	Lesion- or procedure-specific evaluation, rehabilitation history, manual dexterity and support needs	Avoids extrapolation from nerve-sparing prostatectomy evidence to all neurogenic ED.
3. Specialist or exploratory	Advanced testing	Nocturnal penile testing, specialized hemodynamic testing, or other investigations for a defined diagnostic question	Specialist use only; should not delay an established treatment preferred by the patient.
3. Specialist or exploratory	Biomarkers and prediction tools	hs-CRP or other proposed biomarkers; author-proposed response domains or scores	Research use. Candidate predictors have been studied—polymorphisms in PDE5A, NOS3 and VEGF, and nocturnal tumescence and audiovisual stimulation parameters—but none are externally validated for identifying persistent oral non-response or for selecting treatment [97,98].

**Table 6:** What this review adds relative to the overlapping literature. The table is restricted to sources that address management after an inadequate response to a PDE5 inhibitor; it is a positioning statement rather than a systematic comparison.

Source	What It Covers	What It Does Not Do	What This Review Adds
International consultation recommendations [1]	Graded recommendations, including titration to the maximum tolerated dose and offering injection, vacuum device or prosthesis when oral therapy fails or is not tolerated.	Does not describe how to establish that an oral trial was adequate, or how to record the resulting treatment status.	An explicit account of trial adequacy and a status label that is separated from etiology.
European guideline [2]	A dedicated subsection on non- and poor-responders: Verify correct use, food effects, patient education, hypogonadism, and a possible change of agent.	Does not set out how the individual elements relate to each other, and does not state the necessary evidence.	The negative claims stated explicitly, and the elements organized as distinct conceptual axes.
Earlier non-responder reviews [6,9,11]	Established causes of apparent failure, re-education, comorbidity assessment and non-oral options, over two decades.	Earlier reviews do not consistently distinguish conversion in the full referred cohort from conversion among selected patients who accepted re-education.	Reports both 88/250 in the full referred cohort and 88/115 among selected men who accepted re-education [7], while explicitly stating the selection and generalizability limitations.
Contemporary 2026 syntheses [12,49] and the 2026 comprehensive review [14]	Established and newer therapies after PDE5-inhibitor failure and contemporary management of erectile dysfunction overall.	Treats the reassessment step briefly, as a preliminary to second-line treatment.	Reassessment treated as the substantive question, with the safety classification in Table 3.
2026 Perspective on erectile reserve [13]	A conceptual model of PDE5 inhibitors as initiators, enablers and sustainers, with a proposed two-week daily challenge read as an “Erectile Reserve Test”.	Provides no diagnostic-accuracy data and no response threshold on a validated instrument.	A reasoned disagreement with that proposal, set out in Section 7.4, and a testable alternative for prospective evaluation.

## Data Availability

No new data were created or analyzed in this study. The PubMed search-history export, reference-reconciliation record, targeted source-verification results file, and original-to-revised reference-number crosswalk are available from the author upon request.

## Supplementary Materials

The following supporting information can be downloaded at https://www.mdpi.com/article/10.3390/medicina62081582/s1: Supplementary Method S1: Complete literature-search strategy. Supplementary Method S2: Source-selection and verification method. Supplementary Method S3: GAMER-aligned generative-AI disclosure. Supplementary Method S4: Key-prompt log. The PubMed search-history export, reference-reconciliation record, targeted source-verification results file, and original-to-revised reference-number crosswalk are available from the author upon request.
