# Supplementary material for: Apparent Non-Response to Phosphodiesterase Type 5 Inhibitors in Erectile Dysfunction: A Narrative Review of Structured Reassessment and Management
Source: Medicina (Kaunas). 2026 Aug 18;62(8):1582. doi: 10.3390/medicina62081582 (PMC13515885; doi:10.3390/medicina62081582)
Supplement: Supplementary file 1 [file medicina-62-01582-s001.zip › medicina-4455931-supplementary.pdf]

# SUPPLEMENTARY METHODS & GAMER APPENDIX

Apparent Non-Response to Phosphodiesterase Type 5 Inhibitors in Erectile Dysfunction: A Narrative Review of Structured Reassessment and Management

**Document:** Supplementary Methods S1–S4
**Search update: PubMed/MEDLINE through 7 August 2026**
**AI reporting:** GAMER nine-item disclosure plus key-prompt log
**Prepared: 7 August 2026; updated: 9 August 2026**
**Manuscript:** *Apparent Non-Response to Phosphodiesterase Type 5 Inhibitors in Erectile Dysfunction: A Narrative Review of Structured Reassessment and Management*

**Status of this appendix: Prepared 7 August 2026 and updated 9 August 2026. The electronic search strings and a summary of the AI-use record are reported below.**

## S1. Literature-search strategy

### S1.1. Review design, information sources, and search date

This work was conducted as a narrative review rather than a systematic review or meta-analysis. PubMed/MEDLINE was specified as the principal bibliographic database, with coverage from database inception to the final search date of 7 August 2026. The complete Boolean strings are reproduced in Sections S1.2 and S1.3 so that every search can be re-executed exactly as reported.

The revision workflow also used backward citation checking of relevant guidelines, consensus statements, reviews, and primary studies; targeted identification of directly overlapping publications from 2025–2026; and consultation of current professional-society and regulatory sources. These supplementary sources included the European Association of Urology guidance on sexual and reproductive health, the American Urological Association erectile-dysfunction guidance, the Princeton IV consensus, European Medicines Agency product information, US Food and Drug Administration/DailyMed prescribing information, and jurisdiction-specific summaries of product characteristics where relevant. Manufacturer webpages were not treated as evidence of efficacy, acceptability, adherence, onset, or responder conversion; when used at all, they served only to identify a formulation or regional product, with clinical and safety statements requiring confirmation against peer-reviewed or official regulatory sources.

No language or publication-type filter was embedded in the PubMed strings. Because this was a narrative review, the searches were not used to generate a PRISMA flow diagram or pooled estimate. The PubMed search-history export and the search date are available from the author on request.

### S1.2. Core PubMed/MEDLINE searches

The following Boolean strings define the reproducible core search. Field tags and syntax are shown exactly as intended for PubMed.

### Core search A: apparent failure or non-response

```
("Erectile Dysfunction"[Mesh] OR "erectile dysfunction"[tiab])
AND ("Phosphodiesterase 5 Inhibitors"[Mesh] OR PDE5*[tiab] OR sildenafil[tiab] OR tadalafil[tiab] OR vardenafil[tiab]
OR avanafil[tiab])
AND (nonrespond*[tiab] OR non-respon*[tiab] OR fail*[tiab] OR refractor*[tiab] OR "partial responder"[tiab] OR
"partial response"[tiab] OR salvage[tiab] OR rescue[tiab] OR "inappropriate use"[tiab] OR reeducat*[tiab] OR re-
educat*[tiab])
```

### Core search B: trial adequacy and contributing domains

```
("Erectile Dysfunction"[Mesh] OR "erectile dysfunction"[tiab])
AND ("Phosphodiesterase 5 Inhibitors"[Mesh] OR PDE5*[tiab] OR sildenafil[tiab] OR tadalafil[tiab] OR vardenafil[tiab]
OR avanafil[tiab])
AND (administration[tiab] OR adherence[tiab] OR timing[tiab] OR food[tiab] OR pharmacokinetic*[tiab] OR
stimulation[tiab] OR hypogonad*[tiab] OR testosterone[tiab] OR diabetes[tiab] OR obesity[tiab] OR psychosexual[tiab]
OR partner*[tiab] OR "female sexual dysfunction"[tiab] OR prostatectomy[tiab] OR neurogenic[tiab] OR
vasculogenic[tiab] OR "penile Doppler"[tiab])
```

### Core search C: oral optimization and subsequent management

```
("Erectile Dysfunction"[Mesh] OR "erectile dysfunction"[tiab])
AND ("Phosphodiesterase 5 Inhibitors"[Mesh] OR PDE5*[tiab] OR sildenafil[tiab] OR tadalafil[tiab] OR vardenafil[tiab]
OR avanafil[tiab])
AND (switch*[tiab] OR "daily tadalafil"[tiab] OR combination[tiab] OR testosterone[tiab] OR "vacuum erection"[tiab]
OR intracavernous[tiab] OR alprostadil[tiab] OR prosthes*[tiab] OR shockwave[tiab] OR LiSWT[tiab] OR LI-ESWT[tiab] OR
"cognitive behavioral"[tiab] OR psychotherap*[tiab])
```

### Core search D: safety, contraindications, and adverse effects

```
("Erectile Dysfunction"[Mesh] OR "erectile dysfunction"[tiab])
AND ("Phosphodiesterase 5 Inhibitors"[Mesh] OR PDE5*[tiab] OR sildenafil[tiab] OR tadalafil[tiab] OR vardenafil[tiab]
OR avanafil[tiab])
AND (safety[tiab] OR adverse[tiab] OR myalgia[tiab] OR "back pain"[tiab] OR nitrate*[tiab] OR "nitric oxide
donor"[tiab] OR riociguat[tiab] OR "guanylate cyclase stimulator"[tiab] OR "alpha blocker"[tiab] OR
cardiovascular[tiab])
```

### Core search E: focused 2025–2026 overlap update

```
("erectile dysfunction"[tiab] AND (PDE5*[tiab] OR "phosphodiesterase 5"[tiab]) AND (failure*[tiab] OR
nonrespond*[tiab] OR "erectile reserve"[tiab] OR initiator*[tiab] OR enabler*[tiab] OR sustainer*[tiab]))
AND (2025:2026[pdat])
```

## S1.3. Targeted PubMed/MEDLINE searches

Targeted searches were used to examine topics for which population, comparator, denominator, or claim scope was important to the revision. These searches complemented rather than replaced the core searches.

### Targeted search 1: administration errors, instruction, and re-education

```
("Erectile Dysfunction"[Mesh] OR "erectile dysfunction"[tiab])
AND ("Phosphodiesterase 5 Inhibitors"[Mesh] OR PDE5*[tiab] OR sildenafil[tiab] OR tadalafil[tiab] OR vardenafil[tiab]
OR avanafil[tiab])
AND (misuse[tiab] OR "administration error"[tiab] OR "administration errors"[tiab] OR "inappropriate use"[tiab] OR
education[tiab] OR counselling[tiab] OR counseling[tiab] OR reeducat*[tiab] OR re-educat*[tiab] OR rechallenge[tiab]
OR "FAST approach"[tiab])
```

## Targeted search 2: validated response and patient-reported outcomes

```
("Erectile Dysfunction"[Mesh] OR "erectile dysfunction"[tiab])
AND ("Phosphodiesterase 5 Inhibitors"[Mesh] OR PDE5*[tiab] OR sildenafil[tiab] OR tadalafil[tiab] OR vardenafil[tiab]
OR avanafil[tiab])
AND ("International Index of Erectile Function"[tiab] OR IIEF[tiab] OR IIEF-EF[tiab] OR SHIM[tiab] OR "Sexual Health
Inventory for Men"[tiab] OR "Erection Hardness Score"[tiab] OR EHS[tiab] OR "Sexual Encounter Profile"[tiab] OR
SEP2[tiab] OR SEP3[tiab] OR "Global Assessment Question"[tiab] OR GAQ[tiab])
AND (respon*[tiab] OR outcome*[tiab] OR "clinically meaningful"[tiab] OR "minimal clinically important
difference"[tiab])
```

## Targeted search 3: partner, couple, and female-sexual-function factors

```
("Erectile Dysfunction"[Mesh] OR "erectile dysfunction"[tiab])
AND ("Phosphodiesterase 5 Inhibitors"[Mesh] OR PDE5*[tiab] OR sildenafil[tiab] OR tadalafil[tiab] OR
vardenafil[tiab])
AND (partner*[tiab] OR couple*[tiab] OR dyadic[tiab] OR relationship*[tiab] OR "female sexual function"[tiab] OR
"female sexual dysfunction"[tiab] OR FSFI[tiab] OR "sexual quality of life"[tiab] OR psychosexual[tiab] OR "cognitive
behavioral sex therapy"[tiab])
```

## Targeted search 4: endocrine and cardiometabolic contributors

```
("Erectile Dysfunction"[Mesh] OR "erectile dysfunction"[tiab])
AND ("Phosphodiesterase 5 Inhibitors"[Mesh] OR PDE5*[tiab] OR sildenafil[tiab] OR tadalafil[tiab] OR vardenafil[tiab]
OR avanafil[tiab])
AND (hypogonad*[tiab] OR testosterone[tiab] OR endocrine[tiab] OR diabetes[tiab] OR glycemic[tiab] OR glycaemic[tiab]
OR obesity[tiab] OR "weight loss"[tiab] OR "GLP-1"[tiab] OR "glucagon-like peptide-1"[tiab] OR SGLT2[tiab] OR
"sodium-glucose cotransporter 2"[tiab])
AND (nonrespond*[tiab] OR non-respon*[tiab] OR response[tiab] OR salvage[tiab] OR rescue[tiab] OR combination[tiab])
```

## Targeted search 5: post-prostatectomy and neurogenic erectile dysfunction

```
("Erectile Dysfunction"[Mesh] OR "erectile dysfunction"[tiab])
AND ("Phosphodiesterase 5 Inhibitors"[Mesh] OR PDE5*[tiab] OR sildenafil[tiab] OR tadalafil[tiab] OR vardenafil[tiab]
OR avanafil[tiab])
AND ("radical prostatectomy"[tiab] OR prostatectom*[tiab] OR "nerve sparing"[tiab] OR nerve-sparing[tiab] OR
neurogenic[tiab] OR neuropath*[tiab] OR "spinal cord injury"[tiab] OR "pelvic surgery"[tiab])
AND (response[tiab] OR nonrespond*[tiab] OR rehabilitat*[tiab] OR recovery[tiab] OR washout[tiab] OR
unassisted[tiab])
```

## Targeted search 6: established non-oral treatments and selected adjuncts

```
("Erectile Dysfunction"[Mesh] OR "erectile dysfunction"[tiab])
AND ("Phosphodiesterase 5 Inhibitors"[Mesh] OR PDE5*[tiab] OR sildenafil[tiab] OR tadalafil[tiab] OR vardenafil[tiab]
OR avanafil[tiab])
AND (nonrespond*[tiab] OR non-respon*[tiab] OR fail*[tiab] OR refractor*[tiab] OR "difficult to treat"[tiab])
AND ("vacuum erection device"[tiab] OR "vacuum erection devices"[tiab] OR intracavernosal[tiab] OR
intracavernous[tiab] OR alprostadil[tiab] OR "penile prosthesis"[tiab] OR "penile prostheses"[tiab] OR
shockwave[tiab] OR LiSWT[tiab] OR LI-ESWT[tiab] OR "low intensity extracorporeal shock wave"[tiab])
```

## Targeted search 7: chronic treatment, rehabilitation, and durable unassisted function

```
("Erectile Dysfunction"[Mesh] OR "erectile dysfunction"[tiab])
AND ("Phosphodiesterase 5 Inhibitors"[Mesh] OR PDE5*[tiab] OR sildenafil[tiab] OR tadalafil[tiab])
AND (rehabilitat*[tiab] OR "endothelial rehabilitation"[tiab] OR "tissue conditioning"[tiab] OR fibrosis[tiab] OR
antifibrotic[tiab] OR "disease modifying"[tiab] OR disease-modifying[tiab] OR REACTT[tiab] OR washout[tiab] OR
unassisted[tiab] OR "return to baseline"[tiab])
```

### Targeted search 8: regenerative and immune-cell approaches

```
("Erectile Dysfunction"[Mesh] OR "erectile dysfunction"[tiab])
AND (regenerat*[tiab] OR "immune cell"[tiab] OR "immune cells"[tiab] OR immunocentric[tiab] OR macrophage*[tiab] OR
"mononuclear cell"[tiab] OR "mononuclear cells"[tiab] OR "stem cell"[tiab] OR "stem cells"[tiab] OR "platelet-rich
plasma"[tiab] OR PRP[tiab])
AND (PDE5*[tiab] OR "phosphodiesterase 5"[tiab] OR vasculogenic[tiab] OR nonrespond*[tiab] OR non-respon*[tiab])
```

### Targeted search 9: reporting of generative AI in medical research

```
(("generative artificial intelligence"[tiab] OR "generative AI"[tiab] OR "large language model"[tiab] OR "large
language models"[tiab])
AND (reporting[tiab] OR guideline*[tiab] OR disclosure[tiab])
AND (medical[tiab] OR biomedical[tiab] OR research[tiab]))
OR GAMER[tiab]
```

## S1.4. Search record

All 14 queries defined in Sections S1.2 and S1.3 were executed against PubMed/MEDLINE on 7 August 2026 through the NCBI E-utilities esearch endpoint. The record counts below were captured at the time of execution. Because the searches were run to map the relevant literature for a narrative review rather than to assemble a screened sample, the counts are reported for reproducibility and are not used to construct a PRISMA flow diagram or a pooled estimate.

| Query | Focus | Records retrieved |
|---|---|---|
| A | Core — apparent failure or non-response | 671 |
| B | Core — trial adequacy and contributing domains | 2,533 |
| C | Core — oral optimization and subsequent management | 1,395 |
| D | Core — safety, contraindications and adverse effects | 1,858 |
| E | Core — focused 2025–2026 overlap update | 24 |
| T1 | Targeted — administration errors, instruction, re-education | 197 |
| T2 | Targeted — validated response and patient-reported outcomes | 803 |
| T3 | Targeted — partner, couple and female sexual function | 797 |
| T4 | Targeted — endocrine and cardiometabolic contributors | 305 |
| T5 | Targeted — post-prostatectomy and neurogenic ED | 356 |
| T6 | Targeted — established non-oral treatments and adjuncts | 206 |
| T7 | Targeted — chronic treatment, rehabilitation, unassisted function | 422 |
| T8 | Targeted — regenerative and immune-cell approaches | 169 |
| T9 | Targeted — reporting of generative AI in medical research | 1,927 |

PubMed returned a phrase-not-found warning for two quoted terms: “erectile reserve” in Query E and “endothelial rehabilitation” in Query T7. Both were dropped from the executed query translation. Each was one alternative within an OR set and matched no indexed record, so the retrieved sets are unaffected. The

complete search-history export, including the PubMed query translations and these warnings, is provided as an accompanying file.

### S1.5. Targeted source verification outside PubMed

For reviewer-flagged and other high-risk clinical-guideline, cardiovascular-safety, and regulatory claims, the relevant official EAU, AUA, Princeton IV, EMA, FDA/DailyMed, and national product-information sources were consulted. Recorded access dates for the official online sources cited in the final manuscript range from 7 July to 8 August 2026. Exact-title, author, DOI, PMID, and publisher checks were also used to confirm bibliographic identity and to locate the two directly overlapping 2026 publications. Because product labels and device status may change, the jurisdiction-specific wording reflects the dated official source identified in the corresponding reference entry.

## S2. Source selection, claim verification, and methodological limitations

### S2.1. Source selection

Sources were selected purposively for direct relevance to apparent PDE5-inhibitor non-response, adequacy of treatment exposure, characterization of response, contributing clinical domains, treatment optimization, subsequent treatment, adverse effects, contraindications, and shared decision-making. The following hierarchy guided use of evidence:

1. Current clinical-practice guidelines, consensus statements, and official regulatory documents for recommendations, contraindications, licensed dosing, and regulatory status.
2. Randomized trials and prospective original studies for numerical efficacy, response, and safety claims.
3. Systematic reviews and meta-analyses for synthesis across studies, with the original study retrieved when the exact population, comparator, denominator, or outcome was essential.
4. Large observational studies for uncommon safety outcomes and selected real-world questions, with residual confounding and indication bias stated where relevant.
5. Retrospective, narrative, conceptual, mechanistic, animal, and early translational sources only for clearly delimited exploratory or hypothesis-generating statements.
6. Product or manufacturer sources only for product existence or regional availability; they were not used to infer superiority, adherence, acceptability, clinical onset, or conversion from non-response.

Duplicate reports, irrelevant indications or populations, routine-efficacy claims based only on case reports, and promotional claims lacking peer-reviewed or regulatory support were excluded from the evidentiary basis of the clinical pathway. Direct evidence in men with documented prior PDE5-inhibitor non-response was distinguished from indirect evidence in broader erectile-dysfunction populations, mechanistic/preclinical evidence, guideline position, and regulatory status. These categories are descriptive and are not GRADE ratings.

## S2.2. Selection and verification process

The review was prepared by one author using a structured, purposive narrative-selection process. Generative artificial intelligence was not used for literature searching, source selection, evidence appraisal, or drafting; its use was confined to preparation of the figures, as reported in Section S3.

Bibliographic-identity and DOI/PMID checks were performed on an intermediate 111-entry revision of the reference list; they were not an independent audit of either the 104-reference original submission or the final 117-reference list. Source-level checking was targeted to reviewer-flagged and selected high-risk claims, particularly study design and population, numerator and denominator, comparator, effect direction, follow-up, and regulatory or guideline wording. Preclinical evidence was labelled as such, and selected treatment-assisted outcomes were distinguished from durable unassisted outcomes after withdrawal. This was not a duplicate or comprehensive verification of every retained clinical claim.

Fourteen PubMed/MEDLINE searches were executed on 7 August 2026; their exact queries, PubMed translations, warnings, and retrieved counts are documented in the accompanying search-history export and summarized in Section S1.4. The accompanying reconciliation record states the scope and limitations of the intermediate bibliographic checks, and the accompanying source-verification results file documents 14 targeted numerical and claim-scope checks with both original and revised reference numbers. Selected full texts, the complete 2026 EAU guideline, and relevant official product information were consulted where needed. Targeted checks included re-education denominators, relevant study populations and designs, selected treatment comparators and effect directions, preclinical-versus-clinical inference, and jurisdiction-specific safety and regulatory wording. These checks led to correction or narrowing of the corresponding passages described in the response letter. Because this was a single-author narrative review, no duplicate screening or independent comprehensive audit of all citation markers or sentence–reference pairs was performed; residual citation or transcription errors may remain.

## S2.3. Methodological limitations

This narrative review had no preregistered protocol, duplicate screening, formal study-selection flow diagram, formal risk-of-bias assessment, GRADE assessment, or quantitative synthesis. Search and selection decisions were made purposively rather than through a prespecified systematic-review protocol; selection and citation bias therefore cannot be excluded. The literature is heterogeneous in its definition of non-response, baseline erectile-dysfunction severity, prior treatment exposure, patient population, outcome measure, and duration of follow-up. Several treatment claims derive from general erectile-dysfunction populations rather than confirmed PDE5-inhibitor non-responders. Regulatory and device status varies by jurisdiction and may change over time.

The four-stage structured reassessment framework is an author-proposed educational synthesis. It was not developed through Delphi consensus, external expert consensus, patient or partner involvement, content-validity testing, inter-rater reliability assessment, diagnostic-accuracy testing, or prospective validation. Citations may support individual components, but they do not validate the framework as a whole. The term “residual erectile reserve” is therefore used only as a physiological heuristic and not as a validated diagnostic or prognostic test.

# S3. GAMER-aligned disclosure of generative-AI use

Reporting follows the nine items of the GAMER Statement: general declaration, tool specifications, prompting technique, development of a new model, role in the study, AI-assisted manuscript sections, content verification, data privacy, and impact on interpretation or conclusions.

## S3.1. Nine-item disclosure table

| GAMER item | AI tools and uses | Status |
|---|---|---|
| **1. General declaration** | Generative AI was used in one role only: BioRender AI for the visual drafts of Figures 1 and 2. | Both uses are disclosed in the manuscript, this supplement, and the response letter. |
| **2. Tool specifications and dates** | BioRender AI within the BioRender web application (version/build not displayed), used on 6 August 2026 for Figure 1 and on 9 August 2026 for the final Figure 2, under the corresponding publication licences. | Available tool and date information is reported; unavailable build information is stated. |
| **3. Prompting technique and interaction** | Author-directed natural-language prompts specified the scientific content and the visual hierarchy of each figure. | Section S4 summarizes the principal prompt purposes; it is not a verbatim transcript. |
| **4. New or fine-tuned model developed** | No new generative-AI model was developed, trained, or fine-tuned for either use. | Complete. |
| **5. Role in the study/revision** | BioRender AI supported figure drafting and layout only. It did not perform evidence selection, numerical analysis, or clinical decision-making. | All substantive decisions and final approvals remained with the author. |
| **6. AI-assisted manuscript content** | AI assistance affected Figures 1 and 2 and, indirectly, their captions. No other part of the manuscript was AI-assisted. | Affected content is identified. |
| **7. Content verification and modification** | Scientific accuracy, labels, spelling, arrow logic, caption consistency, and licence attribution were reviewed by the author against the sources and the publication licences. | **All final content was approved by the author; no comprehensive independent AI audit is claimed.** |
| **8. Data privacy and confidentiality** | No patient-level or identifiable clinical data were entered. Inputs were limited to author-directed descriptions of the intended figure content. | No patient-identifiable data were used. |
| **9. Potential impact on interpretation, accuracy, or conclusions** | BioRender AI could affect visual emphasis in the figures. It did not replace the author's final scientific and clinical judgment, and it had no effect on the text, the evidence, or the conclusions. | Final interpretation and conclusions reflect the author's judgment, and the author accepts responsibility. |

## S3.2. Disclosure statement

**Use of generative artificial intelligence. BioRender AI within the BioRender web application (version/build not displayed; BioRender.com) was used to generate the visual draft for Figure 1 on 6 August 2026 and for Figure 2 on 9 August 2026 from author-directed prompts; the author reviewed, edited, and exported the final figures under the corresponding publication licences. This was the only use of generative artificial intelligence. No generative-AI tool was used for literature searching, study selection, evidence appraisal, data extraction, citation assignment, development of the reassessment framework, drafting of the text, or formulation of the conclusions. No patient-level or identifiable clinical data were entered, and no AI system is an author.** All AI-assisted suggestions and document changes were reviewed and approved by the author, who accepts full responsibility for the submitted work.

# S4. Short key-prompt log

This table summarizes the principal purposes of the author-directed prompts used with BioRender AI. It is not a complete verbatim transcript.

| Date | Tool | Key prompt or instruction summary | Output used in the revision |
|---|---|---|---|
| 6 August 2026 | BioRender AI | Create a publication-ready medical figure that separates the NO–cGMP/PDE5 mechanism from administration, exposure, adherence/access, and behavioral/contextual barriers, and shows complete, partial, absent response, or discontinuation as observed outcomes. | Figure 1 visual draft. The entry above is the author's record of the instruction given; BioRender does not export a verbatim prompt transcript. |
| 9 August 2026 | BioRender AI | Create a clean four-step pathway: core reassessment → validated response assessment → targeted evaluation of contributing domains → shared decision between oral optimization and established second-line options; show switching, daily tadalafil, and penile Doppler only when clinically appropriate and persistent oral non-response as a final status. | Figure 2 visual draft. The entry above is the author's record of the instruction given; BioRender does not export a verbatim prompt transcript. The figure was revised by the author through successive drafts, and the final version was re-exported on 9 August 2026 under a new publication licence. |

## Supplementary reference

Luo, X.; Tham, Y.C.; Giuffrè, M.; Ranisch, R.; Daher, M.; Lam, K.; Eriksen, A.V.; Hsu, C.-W.; Ozaki, A.; de Moraes, F.Y.; Khanna, S.; Su, K.-P.; Begagić, E.; Bian, Z.; Chen, Y.; Estill, J.; GAMER Working Group. Reporting guideline for the use of Generative Artificial intelligence tools in MEdical Research: the GAMER Statement. *BMJ Evidence-Based Medicine* **2025**, *30*, 390–400. https://doi.org/10.1136/bmjebm-2025-113825.
